# Solving Max‐Cut Problem Using Spiking Boltzmann Machine Based on Neuromorphic Hardware with Phase Change Memory

**DOI:** 10.1002/advs.202406433

**Published:** 2024-10-23

**Authors:** Yu Gyeong Kang, Masatoshi Ishii, Jaeweon Park, Uicheol Shin, Suyeon Jang, Seongwon Yoon, Mingi Kim, Atsuya Okazaki, Megumi Ito, Akiyo Nomura, Kohji Hosokawa, Matthew BrightSky, Sangbum Kim

**Affiliations:** ^1^ Department of Material Science & Engineering Inter‐University Semiconductor Research Center Research Institute of Advanced Materials Seoul National University Seoul 08826 Republic of Korea; ^2^ IBM Research‐Tokyo Chuo‐ku Tokyo 103‐0015 Japan; ^3^ IBM Thomas J. Watson Research Center Yorktown Heights NY 10598 USA

**Keywords:** Boltzmann machines, combinatorial optimizations, leaky integrate‐and‐fire neurons, max‐cut problems, neuromorphic hardware, spiking neural networks

## Abstract

Efficiently solving combinatorial optimization problems (COPs) such as Max‐Cut is challenging because the resources required increase exponentially with the problem size. This study proposes a hardware‐friendly method for solving the Max‐Cut problem by implementing a spiking neural network (SNN)‐based Boltzmann machine (BM) in neuromorphic hardware systems. To implement the hardware‐oriented version of the spiking Boltzmann machine (sBM), the stochastic dynamics of leaky integrate‐and‐fire (LIF) neurons with random walk noise are analyzed, and an innovative algorithm based on overlapping time windows is proposed. The simulation results demonstrate the effective convergence and high accuracy of the proposed method for large‐scale Max‐Cut problems. The proposed method is validated through successful hardware implementation on a 6‐transistor/2‐resistor (6T2R) neuromorphic chip with phase change memory (PCM) synapses. In addition, as an expansion of the algorithm, several annealing techniques and bias split methods are proposed to improve convergence, along with circuit design ideas for efficient evaluation of sampling convergence using cell arrays and spiking systems. Overall, the results of the proposed methods demonstrate the potential of energy‐efficient and hardware‐implementable approaches using SNNs to solve COPs. To the best of the author's knowledge, this is the first study to solve the Max‐Cut problem using an SNN neuromorphic hardware chip.

## Graphical abstract

This work presents a novel method for solving the Max‐Cut problem using a spiking neural network (SNN)‐based Boltzmann machine (BM), implemented on neuromorphic hardware. The approach demonstrates high accuracy and efficiency, validated through simulations and hardware experiments using a 6‐transistor/2‐resistor (6T2R) neuromorphic chip with phase change memory (PCM) synapses.

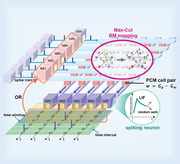

## 1 Introduction

In the emerging era of the Internet of Things and automotive technologies, deciding efficient plans with vast amounts of data from edge and cloud sources is crucial.^[^ ^1^ ^]^ At the core of such decision algorithms lies the foundation of combinatorial optimization problems (COPs), which makes efficient solving of COPs critical. Many COPs are classified as nondeterministic polynomial time hard (NP‐hard) problems, meaning that efficient algorithms for finding their solutions within polynomial time have not been discovered.^[^ ^1^, ^2^ ^]^ As the scale of problems expands, they demand more resources, and their complexity increases.^[^ ^3^ ^]^ Given these considerations, from a practical perspective, it is essential to seek faster and more efficient methods to approximate solutions to COPs. The Max‐Cut problem,^[^ ^4^ ^]^ one of COPs, has applications in VLSI circuit design^[^ ^5^ ^]^ and image segmentation,^[^ ^6^ ^]^ where efficient partitioning can improve performance. Many attempts have been made to solve Max‐Cut; however, a fully parallel and hardware‐oriented algorithm is difficult to achieve.

Spiking neural network (SNN)‐based Boltzmann machine (BM) and neuromorphic computing can be used as effective algorithms for solving fully parallel Max‐Cut problems. BM, one of the energy‐based models, can explore complex energy landscapes, find the energy minimum,^[^ ^7^ ^]^ and stochastically determine subsequent node states using the sigmoid function.^[^ ^4^ ^]^ BM offers a flexible approach, enabling the use of heuristic methods in fast operation time,^[^ ^8^, ^9^ ^]^ making BM an invaluable approach for solving COPs. Neuromorphic computing inspired by the human brain system is a field that is gaining attention, providing powerful approaches to various applications including event‐driven algorithms^[^ ^10^ ^]^ and COPs.^[^ ^11^ ^]^ Neuromorphic computing allows efficient implementation of BM,^[^ ^12^ ^]^ addressing the limitations of conventional computing by emulating neurons and synapses in the human brain.^[^ ^13^ ^]^ Non‐Von Neumann architecture of neuromorphic computing allows substantial parallelism,^[^ ^14^ ^]^ especially in repetitive multiply–accumulate operations in BMs. However, even on a neuromorphic computing scheme, for asynchronous state update, conventional methods based on BM or other energy‐based models require selecting one or some nodes to state update, not allowing fully parallel state update.^[^ ^5^, ^15^, ^16^ ^]^ Applying SNNs with asynchronous firing in neuromorphic computing not only enables fully parallel operation without selecting some nodes to state updates but also enhances energy efficiency from a hardware implementation perspective. SNNs that operate on the principle of spike‐based information processing^[^ ^17^, ^18^ ^]^ exhibit asynchronous and event‐driven operations. In addition, the state transition dynamics of SNNs offer advantages in bypassing high‐energy barriers in COPs,^[^ ^19^ ^]^ thereby presenting a viable, efficient solution for solving COPs. Also, it is well established that the probabilistic nature of SNNs can effectively emulate the stochastic operations characteristic of BM,^[^ ^20^ ^]^ which allows the application of BM‐inspired approaches to SNN‐based computing. Along with other efficient approaches, an analog neuromorphic computing system can significantly enhance the mapping of Max‐Cut problems. Given the varying weight values in Max‐Cut, the need for an analog device capable of representing multiple states is evident. In this context, crossbar arrays with emerging memories have garnered attention as potential analog devices^[^ ^21^ ^]^ such as phase change memory (PCM).^[^ ^22^, ^23^ ^]^ Our previous work demonstrated the feasibility of constructing a neuromorphic chip that incorporates an SNN system using PCM devices.^[^ ^24^, ^25^ ^]^

In this study, we first demonstrate the spiking BM (sBM) by illustrating that the behavior of stochastic leaky integrate‐and‐fire (LIF) neurons with random walk noise mirrors that of BM neurons. This substantiates our choice to use the LIF neuron model and adopt sBM, laying the groundwork for setting the method that we will describe later. We then introduce novel hardware‐friendly methodologies for solving the Max‐Cut using the time window within the sBM framework. We validate the performance of these methods through simulation, showing that sampling convergence is achieved without the need for explicitly selecting specific neurons for state updates. In addition, our experiments on neuromorphic hardware equipped with PCM synaptic arrays confirm the feasibility of the proposed approach, demonstrating its successful application. Furthermore, considering the properties of the neuron model used and SNN, we suggest additional techniques, including two annealing methods and a bias split method, to enhance the performance of Max‐Cut solving. Finally, we propose new circuit designs using PCM synaptic arrays and SNN‐based neuromorphic architecture, specifically engineered to calculate the cut value and identify unstable neurons for efficacious evaluation of convergence.

## 2 Results and Discussion

### 2.1 Construction of sBM Architecture

#### 2.1.1 Neural Sampling

To implement an sBM that solves the Max‐Cut problem, the sBM needs to be guaranteed that it operates analogously to the conventional BM while incorporating the temporal dynamics of neurons. In Buesing et al.,^[^ ^26^ ^]^ the neural sampling theory was proposed to interpret stochastic firing dynamics of SNNs as a nonreversible Markov chain that performs probabilistic inference. A BM can be viewed as a Markov chain that samples from the Boltzmann distribution of given energy defined by its weight and bias. By applying neural sampling theory, we construct a spiking version of the conventional BM, where the firing probability of each neuron is related to its membrane potential, and the state of the network represents a sample from a joint probability distribution defined by the network architecture. Before describing the detailed construction of the proposed model, we summarize the conditions under which neural sampling theory holds.

Consider a neural network composed of N neurons, each characterized by a membrane potential *u*, which governs the dynamic behavior of neurons. A neuron fires based on a probability function linked to its membrane potential. Upon firing, the neuron enters a refractory period that persists for a time τ_ref_ during which its firing probability is zero. The states of neurons denoted as *x*_1_, *x*_2_, …, *x*_N_ are binary: 1 during the refractory period and 0 otherwise. The collective state of the network, represented by vector $x\;=\left(x_{1},\;x_{2},\;\text{…},\;x_{N}\right)$, can be interpreted as a sample from a specific joint probability distribution *p*(*x*), which is described by the connection weights and biases of the network. With these state values, the membrane potential *u*_k_ of neuron *k* can be defined as follows:

(1) $$u_{k}\left(t\right)-\;u_{\mathrm{rst}}=T\cdot log\left(\frac{p\left(x_{k}\left(t\right)\;=\;1|x_{-k}\left(t\right)\right)}{p\left(x_{k}\left(t\right)\;=\;0|x_{-k}\left(t\right)\right)}\right)$$

where *u*_k_ denotes the membrane potential of neuron *k*, *x*_k_ denotes the binary states of neuron *k*, *x*_−k_ denotes all current neuron states, excluding the state of neuron *k*, *u*_rst_ denotes the reset potential indicating a membrane potential without any input, and *T* represents the temperature‐like scaling factor. This is called the neural computability condition. From this, the instantaneous firing rate of neuron *k*, which is derived from neural sampling theory, can be defined as follows:

(2) $$\rho \left(u_{k}\left(t\right)\right)=\;\left\{\begin{array}{c} \;0\;\mathrm{if}\;t-t_{k}^{\mathrm{last}}\;<\;\tau_{\mathrm{ref}}\\ \tau_{\mathrm{ref}}^{-1}\cdot \exp\left(\frac{u\left(t\right)-u_{\mathrm{rst}}}{T}\right)\;\mathrm{if}\;t-t_{k}^{\mathrm{last}}\;>\;\tau_{\mathrm{ref}} \end{array}\right.$$

where $t_{k}^{\mathrm{last}}$ denotes the time of the last spike in neuron *k*.

To model the network as a BM, we set the joint probability distribution *p*(*x*) to the Boltzmann distribution as follows:

(3) $$p\left(x\right)=\frac{1}{Z}\exp\left(\frac{\Sigma_{i,j}w_{\mathrm{ij}}x_{j}+\;\Sigma_{i}b_{i}x_{i}}{T}\right)$$

where *w*_ij_ denotes the synaptic weight between neurons *i* and *j*, *b*_i_ denotes the bias of neuron *i*, and *Z* represents the partition function ensuring normalization. Note that self‐connections are excluded (*w*_ii_ = 0).

Using Equation (3), we can compute the conditional probability distribution in Equation (1) and derive the exact membrane potential *u*_k_(*t*) as follows:

(4) $$\begin{array}{ccc} u_{k}\left(t\right) & = & \;u_{\mathrm{rst}}+b_{k}+\Sigma_{l=1}^{N}w_{\mathrm{kl}}x_{l}\left(t\right)=u_{\mathrm{rst}}+b_{k}\\ & & +\Sigma_{l=1}^{N}w_{\mathrm{kl}}\Sigma_{s=1}^{S}\Theta \left(t-t_{k}^{s}\right)\Theta \left(t_{k}^{s}-t+\tau_{\mathrm{ref}}\right) \end{array}$$

where Θ denotes the Heaviside function, and $t_{k}^{s}$ denotes the timing of the s‐th spike for neuron *k*.

The neuron model constructed under the above neural sampling conditions is called the Abstract neuron model in the sBM context.

#### 2.1.2 Neuron Model for Hardware and Software Simulations

Implementation of the Abstract neuron model, which corresponds to Gibbs sampling in an equivalent BM with identical weights and biases, is well‐supported by neural sampling theory. Despite its theoretical robustness, realizing this model in a hardware framework poses significant challenges. We describe two reasons why hardware implementation is difficult and how our neuron model addresses these issues.

##### Complex Postsynaptic Potential (PSP) Dynamics

The proposed PSP $\Sigma_{s\;=\;1}^{S}\Theta \left(t-t_{k}^{s}\right)\Theta \left(t_{k}^{s}-t+\tau_{\mathrm{ref}}\right)$ in Equation (4) assumes a rectangular form. Such a shape is challenging to implement in hardware systems that rely on capacitors to store membrane potentials.

In practice, PSPs can be modeled by various functions, allowing membrane potentials to be expressed as a convolution of the input spike train with an arbitrary PSP kernel κ, as shown in Equation (5).

(5) $$u_{k}\left(t\right)=u_{\mathrm{rst}}+b_{k}+\Sigma_{l\;=\;1}^{N}w_{\mathrm{kl}}\Sigma_{s\;=\;1}^{S}\kappa \left(t-t_{k}^{s}\right)$$

Thus, alternative PSP shapes, such as exponential ($\kappa \left(s\right)=\;A\cdot e^{-\frac{s}{\tau_{1}}})$, biexponential $\left(\kappa \left(s\right)=A\cdot \left(e^{-\frac{s}{\tau_{1}}}-e^{-\frac{s}{\tau_{2}}}\right)\right)$,^[^ ^20^, ^26^ ^]^ and alpha $\left(\kappa \left(s\right)=\;A\cdot s\cdot e^{-\frac{s}{\tau }}\right)$ PSPs, can be used to substitute rectangular PSPs. These shapes are not only feasible for hardware implementation but also considered more biologically plausible according to the neuroscience literature.^[^ ^27^ ^]^

In our hardware system, we opted for an exponential kernel because of its simplicity and compatibility with hardware implementation. The exponential decay characteristic is effectively replicated using the LIF model, which equivalently models RC(resistor–capacitor) circuits. ($C\frac{d}{dt}u\;=\;-g\left(u-\;u_{\mathrm{rst}}\right)\;+\;I\left(t\right)$, where *C* represents the membrane capacitance, *g* represents the leak conductance, and *I*(*t*) denotes the input current to the neuron at time *t*.) Furthermore, as described in a later section, although several COPs are tractable using the Abstract neuron model,^[^ ^19^ ^]^ in our Max‐Cut solving algorithm, the LIF model appears to outperform the rectangular PSP model because of the smoothing of neuronal state changes.

##### Stochastic Firing Mechanism

The spike firing mechanism of the Abstract neuron model is inherently probabilistic, with the firing rate varying according to the membrane potential, as explained in Equation (2). However, the direct implementation of varying probabilities at the device level is strenuous. Although several devices have been developed for simulating stochastic behavior by directly embodying probabilities using inherent noisy dynamics in various physical systems,^[^ ^28^, ^29^ ^]^ high‐precision mapping between the desired inputs and probabilistic outputs is difficult because of inherent randomness, which makes consistency across multiple devices difficult.

To address these issues, we introduce an alternative stochastic neuron model that does not rely on direct calculations of firing probabilities. Computational neuroscience has presented two primary models for stochastic neurons: the escape noise model and the diffusive noise model.^[^ ^30^ ^]^ The former, similar to the Abstract neuron model discussed earlier, uses a variable firing rate to determine neuron activation. The latter, our model of choice, encapsulates continuous random fluctuations in the membrane potential, mirroring the random arrival of synaptic inputs. In the diffusive noise model, a threshold is set, and firing occurs when the membrane potential exceeds this threshold, thereby integrating a deterministic element into the stochastic firing process, which simplifies the hardware implementation. We achieve this by modulating the membrane potential with random walk noise. Firing is determined using a comparator, and a random walk is generated using standard random number generation circuits. For each random walk clock timing of the interval *t*_step_, the membrane potential is adjusted upward (*v*_up_) or downward (*v*_dn_) with an equal probability, maintaining the stochastic nature of the model.

The proposed neuron model for hardware combines an exponential PSP with random walk‐induced diffusive noise and is encapsulated in a LIF model framework. The proposed model can be mathematically represented as follows:

(6) $$\tau_{\mathrm{leak}}\frac{d}{\mathrm{dt}}u_{k}=-\left(u_{k}-u_{\mathrm{rst}}\right)+I\left(t\right)+\xi ,u_{k}\left(t\right)\in \left(-\infty ,\theta \right)$$

where ξ denotes the noise induced by random walk. When the membrane potential *u* exceeds θ, it is reset to *u*_rst_. Note that the LIF model has a different refractory mechanism compared to the Abstract neuron model. In the LIF model, the membrane potential is reset, and no input is received during the refractory period, whereas in the Abstract neuron model, the membrane potential is not reset, and input is still received during the refractory period. To infer the Max‐Cut state within the given sBM architecture, the input *I*(*t*) is modulated as follows:

(7) $$I\left(t\right)\propto b_{k}+\Sigma_{l\;=\;1}^{N}w_{\mathrm{kl}}M\left(t,l\right)\Sigma_{s\;=\;1}^{S}\delta \left(t-t_{k}^{s}\right)$$

where *M*(*t*, *l*) denotes the mask function, which is set to 1 if the *l*‐th neuron fired at least once in the preceding interval and 0 otherwise; this will be explained more in the Max‐Cut solving method section; $t_{k}^{s}$ denotes the spike timing of the input spike file generated by a specific stochastic process. We used the Poisson process with dead time (PPD) to generate input spikes. For brevity in hardware implementation, the input is a spike train represented by the sum of the delta function in Equation (7).

For software simulation, Equation (6) was developed to first order using Euler's method.

(8) $$\begin{array}{ccc} & & u\left(t+t_{\mathrm{step}}\right)=\\ & & \left\{\begin{array}{c} \left(1-\frac{t_{\mathrm{step}}}{\tau_{\mathrm{leak}}}\right)u\left(t\right)+\frac{u_{\mathrm{rst}}t_{\mathrm{step}}}{\tau_{\mathrm{leak}}}+\frac{t_{\mathrm{step}}}{\tau_{\mathrm{leak}}}I\left(t\right)+v_{\mathrm{up}},\;\mathrm{by}\;50\%\;\mathrm{prob}\mathrm{abil}\mathrm{ity}\\ \left(1-\frac{t_{\mathrm{step}}}{\tau_{\mathrm{leak}}}\right)u\left(t\right)+\frac{u_{\mathrm{rst}}t_{\mathrm{step}}}{\tau_{\mathrm{leak}}}+\frac{t_{\mathrm{step}}}{\tau_{\mathrm{leak}}}I\left(t\right)-v_{\mathrm{dn}},\;\mathrm{by}\;50\%\;\mathrm{prob}\mathrm{abil}\mathrm{ity} \end{array}\right. \end{array}$$

For the hardware implementation model, the Diffusive LIF neuron model with an exponential PSP and random walk noise described above was used. For software simulation, the Abstract neuron model with a rectangular PSP and escape probability, the escaping LIF neuron model with an exponential PSP and escape probability,^[^ ^31^, ^32^, ^33^ ^]^ and the Diffusive LIF neuron model were used for comparison as ideal, mixed, and hardware cases, respectively.

To ensure a fair comparison between the three models, their parameters were calibrated to match each other. The calibration process involves adjusting the scaling factor of the exponential PSP model based on the scaling factor of the rectangular PSP model using parameters. τ_leak_ and τ_ref_ and calculating parameter *T* in the escape noise model using parameters *v*_up_ and *v*_dn_ from the Diffusive noise model (**Table** **1**). To calibrate the scaling factor of the exponential PSP model, we calculated and compared the stationary membrane potential with different PSPs. To calibrate parameter *T* in the escape noise model based on parameters *v*_up_ and *v*_dn_ in the Diffusive noise model, we derive the equation for the average firing rate ρ in terms of the stationary membrane potential, i.e., the inverse of the sum of the refractory time and the average time spent for the first spike immediately after the refractory period, and we fitted the plots for different models. The detailed calibration process is described in Section S1 (Supporting Information), and the stationary membrane potential and average firing rate are defined in Sections S2 and S3 (Supporting Information).

#### 2.1.3 Verification of Neural Sampling Capability

**Figure 1:**
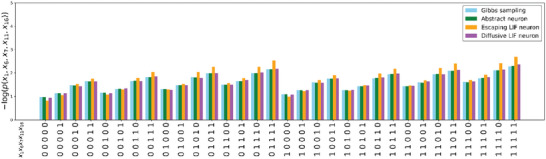
Comparison of Gibbs sampling result and neural sampling results from the three different neuron models. BM consists of 30 nodes, and the weights and biases of BM are sampled from *N*(0,  0.03) and *N*(− 1.5,  0.75), respectively. A total of 10^7^ samples were obtained for each sampling method within a simulation time of 100 s. A Burn‐in time of 10 s was applied at the beginning of the simulation to ensure accurate sampling results. The negative log of marginal probabilities for five randomly selected nodes is calculated and plotted above.

To evaluate the effectiveness of the calibration, it is essential to verify that the models can accurately sample the target Boltzmann distribution defined by the Max‐Cut problem parameters. We tested this concept using a 30‐node BM using Gibbs sampling and a 30‐node sBM with three different neuron models for neural sampling. All networks had the same weights and biases, randomly selected from a normal distribution. We present the sampling results of five randomly selected nodes for each model in **Figure** **1**. The Abstract neuron model achieved results comparable to those of the Gibbs sampler because it is mathematically proven to sample directly from the exact target Boltzmann distribution. Although the Escaping and Diffusive LIF models exhibited more variability in their results than the ideal Abstract model, the differences were not statistically significant, affirming a successful calibration. These findings align with previous studies that employed bi‐exponential PSP models.^[^ ^20^, ^26^ ^]^

**Table 1:** Summary of the three neuron models. PSPs shape, neuron model, and corresponding adjustable parameters for each neuron model.

Model	PSP shape	Stochastic firing model	Adjustable parameters
Abstract neuron model	Rectangular PSPs (width is τ_ref_)	Escape rate	τ_ref_, *u*_rst_, *T*, PSP amplitude
Escaping LIF neuron model	Exponential PSPs		τ_ref_, *u*_rst_, τ_leak_, *T*, PSP amplitude
Diffusive LIF neuron model		Diffusive noise (random walk)	τ_ref_, *u*_rst_, τ_leak_, *t*_step_, *v*_up_, *v*_dn_, *T*, PSP amplitude

### 2.2 Solving the Max‐Cut Problem in sBM Framework

#### 2.2.1 Max‐Cut Problem sBM Mapping

**Figure 2:**
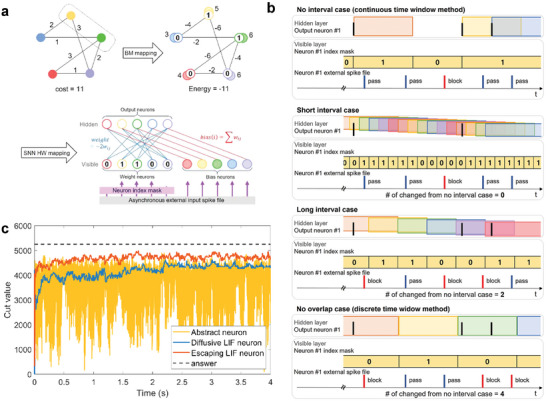
Hardware‐oriented time window overlap method for the Max‐Cut problem. a) example of the Max‐Cut problem and BM mapping of the problem. SNN hardware implementation scheme for solving Max‐Cut. b) Delayed state update to neuron index mask and different masking of spike files depending on the length of time interval. From top to bottom, no interval case (continuous time window method), time window overlap method with short intervals, time window overlap method with large intervals, and no overlap case (discrete time window method). The spike files colored red indicate blocked spikes by the neuron index mask, and blue spike files are passed by the neuron index mask. Masking of spike files in the short interval case is the same with no interval case, but the large interval case shows two differences and no overlap case shows four differences with no interval case as time intervals widen. c) Simulation results of the Max‐Cut problem with the Abstract neuron, Diffusive LIF neuron, and Escaping LIF neuron models. The time window length is set as τ_ref_ for the Abstract neuron model, and 2τ_ref_ for the Diffusive LIF neuron, Escaping LIF neuron models. This problem is made by Rudy^[^ ^35^ ^]^ benchmarking other previous papers. The Max‐Cut problem used is a weighted Max‐Cut problem where the weights are integers ranging from 1 to 10, having 100‐node with 30% weight density. The x‐axis is time in simulation and the *y*‐axis is a calculated cut value from the neuron index mask at the end of every time window. The black dashed line is the answer to the problem gained from BiqMac (binary quadratic and Max‐cut) solver.^[^ ^36^ ^]^

The Max‐Cut problem is expressed on an undirected graph comprising *N* nodes represented by a symmetric weight matrix.^[^ ^4^, ^34^ ^]^ This problem involves partitioning the nodes of the graph into two partitions, *V*1 and *V*2, to maximize the sum of the weights of the edges that cross the partitions.^[^ ^4^ ^]^ An illustrative example of the Max‐Cut problem is shown in **Figure** **2**a. In this scenario, the weight between the *i*‐th and *j*‐th nodes is denoted as *w*_ij_, and the state of the *i*‐th node, *x*_i_, is set to 1 if the node belongs to partition *V*1 and 0 if it belongs to partition *V*2. The cost function of the Max‐Cut problem is the sum of *w*_ij_ for edges between nodes in different partitions.^[^ ^12^ ^]^ The cost function rearranged to resemble the form with the energy function of BM is as follows:

(9) $$cost\;function=\sum_{i\;=1}^{N}\left(\sum_{j\;=\;1}^{N}w_{\mathrm{ij}}\right)\cdot x_{i}+\sum_{i,j}\left(-2w_{\mathrm{ij}}\right)\cdot x_{i}\cdot x_{j}$$

The goal is to find the state vector $x_{1},\;x_{2},\;\text{…},\;x_{N}$ that maximizes this cost function.^[^ ^4^, ^34^ ^]^ Figure 2a illustrates how the Max‐Cut problem can be mapped onto a BM. A comparison between the cost function of the Max‐Cut problem (Equation (9)) and the energy function of the BM (Equation (10)) leads to the derivation of the weight and bias equations for the BM representation of the Max‐Cut problem:^[^ ^12^, ^34^ ^]^

(10) $$energy\;function=-\left(\sum_{i,j}d_{\mathrm{ij}}\cdot x_{i}\cdot x_{j}+\sum_{i}\theta_{i}\cdot x_{i}\right)$$

(*d*_ij_ is weight, θ_i_ is the bias of energy function)

(11) $$weight\left(i,j\right)=-2w_{\mathrm{ij}}$$

(12) $$\mathrm{bias}\left(i\right)=\sum w_{\mathrm{ij}}$$

#### 2.2.2 Max‐Cut Mapping on SNN Hardware

The Max‐Cut problem in BMs can be implemented on SNN‐based neuromorphic hardware, as shown in Figure 2a. This configuration adopts a bilayer structure comprising a visible layer for generating input spikes and a hidden layer for firing output spikes to facilitate hardware implementation by separating the input and output layers. Within this structure, the nodes from the Max‐Cut problem are mapped to three types of neurons: weight neurons in the visible layer representing input spikes representing neuron states; bias neurons in the visible layer representing bias spikes; output neurons in the hidden layer receiving input and firing output spikes. Theoretically, biases should be applied directly to adjust the resting potential of neurons; however, to facilitate bias implementation in hardware, we implemented biases as spike trains with firing rates equal to those of the input spike trains. This approach allows us to use the same mechanism that generates input spikes for bias spikes, thereby eliminating the need for separate hardware components to adjust the rest potential and simplifying hardware design. The deviation from ideality was well addressed by the bias split method, as explained in the following section. The connections between neurons are aligned using Equations (11) and (12), ensuring that weights are symmetric and that *weight*(*i*,  *i*) is zero. During sampling, forward input spikes from the visible layer to the hidden layer are used, and state updates are repeated to weight neurons using the results from the output neurons. Although this bilayer structure resembles a restricted BM, it serves only as a hardware mapping strategy rather than a direct implementation. Our operational approach aligns with that of a BM, and the subsequent explanations are based on the scheme illustrated in Figure 2a.

#### 2.2.3 State Representation by Neuron Index Mask

In our implementation of the sBM for solving the Max‐Cut problem, we employ a mechanism involving external input spike files and a neuron index mask (mask function), which indicates the states of weight neurons in the visible layer. This mechanism facilitates hardware‐friendly approaches. This setup allows for the dynamic management of input states by selectively blocking or permitting the firing of prepared external input spikes, as illustrated in Figure 2a. Specifically, when the state of a weight neuron is set to 0 (x_i_ = 0), the corresponding neuron index mask prevents external spikes from being sent to the hidden layer. Conversely, if x_i_ is set to 1, the mask allows external spikes to pass through and send spikes to the hidden layer. The external input spikes are designed to occur asynchronously, characterized by PPD.

#### 2.2.4 State Decision by Time Window Strategy

In traditional BMs, the calculation of the weighted sum of each node (Equation (13)) is based on the states and weights of connections in every iteration. Subsequently, the next states of nodes are probabilistically determined, where the activation function determines a transition to state 1, as described in Equation (14).

(13) $$\mathrm{weig}\mathrm{hted}\;\mathrm{sum}\left(i\right)=\sum_{j}\left(-2w_{\mathrm{ij}}\right)\cdot x_{j}+\sum_{j}w_{\mathrm{ij}}$$

(14) $$P_{\;x_{i}=\;1}=\frac{1}{1+exp\left(-\frac{weighted\;sum\left(i\right)}{T}\right)}\;$$

Our study, which addresses the Max‐Cut problem by utilizing the sBM framework with temporal dimension, introduces a time window to determine neuron states. The sBM strategy for solving the Max‐Cut problem uses a time window to determine neuron states based on the firing rate, and a neuron state is designated as 1 if the corresponding output neuron fires at least once within the time window and 0 if it does not.

The length of the time window is linked to the firing dynamics of the neuron, ensuring that the likelihood of state transitions aligns with the underlying principles of BM. In BM, the probability of being in state 1 and the probability of being in state 0 is determined based on Equation (14). According to neural sampling theory, for the Abstract neuron model, the time window should be set to match the refractory period (τ_ref_) to ensure sampling from the given Boltzmann distribution. However, the firing rate of the Diffusive LIF neuron model is generally lower compared to the Abstract neuron model as shown in Figure S3 (Supporting Information). This lower firing rate poses a problem when using the time window method to determine the neuron's state as 1 or 0, leading to deviations from accurate sampling from the Boltzmann distribution. To address this issue, instead of using the τ_ref_​ from the ideal model, the time window was extended for the LIF neuron models. Experimentally, a time window of 2τ_ref_ was used to compensate for the lower firing rate.

#### 2.2.5 Time Window Overlap Method for Asynchronous State Update

In addressing COPs, many existing methods rely on discrete‐time Hopfield neural network (DHNN) frameworks, which conventionally update one node at a time in each iteration because of DHNN limitations.^[^ ^8^ ^]^ This approach fails to fully exploit the advantages of parallel computing. In other words, although some existing methods update partial neuron states at a time using update patterns or schedules, they require preparation or repeating the same patterns,^[^ ^16^ ^]^ not fully exploiting parallel computing. In contrast, our research overcomes these drawbacks by employing an innovative update scheme that uses asynchronous spiking and LIF neurons in the sBM framework. A key contribution of this study is the time window overlap method, which introduces time windows and orchestrates their overlapping to check and update neuron states dynamically without the additional process of selecting specific neurons to update the state.

The time difference between time windows is defined as a time interval, which allows for the strategic overlap of time windows by specific intervals, as shown in Figure 2b. The most asynchronous and instantaneous state update scheme in this framework is the no‐interval case (continuous time window method), where neuron index masks are updated instantly with each spike generated from the output neurons, indicating an extremely short or no time interval between updates, thereby promoting a nearly continuous operation. However, achieving true continuity is impractical in hardware systems that are subject to clock cycles and computational constraints. Extremely short intervals, which lead to frequent updates, can significantly increase circuit operation and computational load. Conversely, in the no‐overlap case (the discrete time window method), where the time windows do not overlap (Figure 2b), synchronized updates of asynchronous output spikes are observed at the end of each time window, which potentially reduces the accuracy of the search due to delayed and synchronized state updates.

We propose an optimal interval length that considers the number of nodes in the problem and balances the two extreme cases. The complexity of the energy landscape and the number of spiking output neurons increase with additional nodes; thus, the number of nodes is considered. Therefore, we set the time interval length to 2τ_ref_ divided by the number of nodes. This time interval length can facilitate efficient state updates and accurate masking of spike files. This compromise between the no‐interval and no‐overlap cases optimizes search accuracy while maintaining a manageable computational workload and hardware operation efficiency.

#### 2.2.6 Overall Procedure and Convergence Criteria

We begin sampling with uniform neuron index masks for all neurons and set the initial cut value to 0. As the simulation progresses, the neuron index mask is dynamically updated at the end of each time window, reflecting the firing activity of output neurons within the window. In the visible layer, the mask is adjusted to block or pass the prepared spike files. This iterative process, repeated in every subsequent time window, facilitates the search for the optimal solution by continuously sampling and evaluating the network state.

To assess the convergence of our sampling method, we primarily employ the cut value which can be calculated with cost function (Equation (9)) as a criterion, which has been used in previous studies^[^ ^5^, ^15^ ^]^ to demonstrate sampling convergence. In this study, we indicated the solutions from the BiqMac^[^ ^36^ ^]^ solver alongside the cut value graphs, allowing us to observe how quickly and how closely the sampling converges to the solution. In addition, we propose another criterion that focuses on the number of unstable neurons, i.e., neurons that are more likely to change states than to remain static. This likelihood is inferred from the weighted sum of each neuron, as established in BM theory, which indicates the predominant state (1 or 0). Unstable neurons are identified by comparing their weighted sum (Equation (13)) with their current states, which allows us to discern potential state transitions. The conditions for unstable neurons are as follows:

(15) $$\left\{\begin{array}{c} \mathrm{weig}\mathrm{hted}\;\mathrm{sum}\left(i\right)>0\;\mathrm{if}\;x_{i}=0\\ \mathrm{weig}\mathrm{hted}\;\mathrm{sum}\left(i\right)<0\;\mathrm{if}\;x_{i}=1 \end{array}\right.$$

Notably, the concept of unstable neurons introduces an additional metric to evaluate system dynamics and provides a practical stopping criterion to solve the Max‐Cut problems in scenarios where the exact answer is unknown.

#### 2.2.7 Max‐Cut Simulation Results for Three Neuron Models

First, simulations are performed to compare the three neuron models referred to earlier: the Abstract, Escaping LIF, and Diffusive LIF neurons. Figure 2c shows the simulation outcomes for the neuron models. The Abstract neuron model exhibited the most pronounced fluctuations among the three models, because of its rectangular PSP, which leads to numerous abrupt changes in the membrane potential, causing severe fluctuations in the neuron index masks or neuronal states. This instability can be attributed to the time intervals, which are too short for the membrane potentials of neurons to fully adjust to state changes. Also, Since the Abstract model lacks a reset mechanism in the refractory period, if the network's state is changed at short intervals based on the time window, there is insufficient time for the system to converge from the stationary potential of the previous state to that of the next state. Consequently, the results may not indicate accurate samplings.

Conversely, the Escaping LIF neuron model exhibited significantly more stable behavior, with convergence near the optimal solution and minimal fluctuations, effectively smoothing neuronal state transitions. This smoothing action bridges the gaps between time intervals, ensuring more consistent and stable convergence in solving the Max‐Cut problem. However, despite minimal fluctuations with stable behaviors of neurons, the sampling is not trapped in local minima, utilizing sampling time efficiently. It is due to the spike‐based dynamics which bypass the higher energy barrier.^[^ ^19^ ^]^ This result suggests that the LIF neuron model is well suited to the time window overlap method in the sBM architecture. However, the hardware implementation of the Escaping LIF neuron presents a significant challenge in terms of calculating the firing probability depending on membrane potentials despite its superior performance.

Given these considerations, the Diffusive LIF neuron model emerges as a feasible and efficient alternative to our methodology. The Diffusive LIF neuron model also achieves results comparable to those of the Escaping LIF neuron model, as depicted in Figure 2c, showing stable convergence while continuing to explore. The proposed model offers a practical balance between performance and ease of hardware implementation, making it an optimal choice for use in the proposed method. Therefore, the Diffusive LIF neuron model is used in subsequent simulations, as described in the next section. In addition, the sampling time in these simulations can vary depending on different time scales, including the refractory period and random walk injection time.

#### 2.2.8 Max‐Cut Hardware Experiments on Neuromorphic Hardware

**Figure 3:**
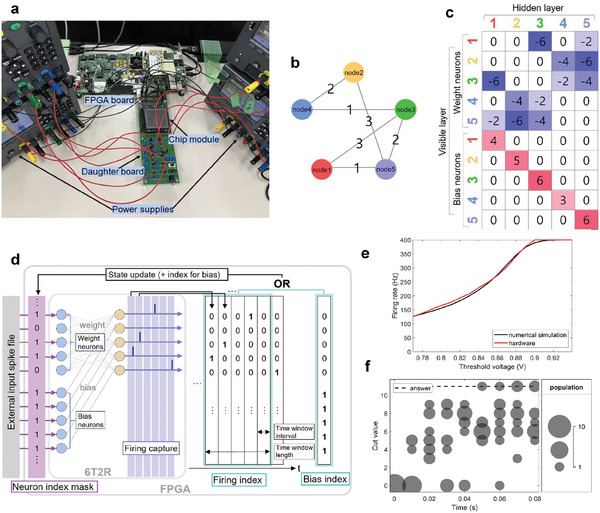
Hardware implementation of time window overlap method on SNN‐based 6T2R neuromorphic chip. a) FPGA‐equipped SNN chip. b) 5‐node Max‐Cut problem for hardware experiments. c) Target weight and bias matrix corresponding to (a). d) hardware experiment scheme on SNN chip and FPGA. e) the average firing rate by a random walk of stochastic LIF neurons in the 6T2R chip. f) bubble chart of experiment results solving the Max‐Cut on SNN chip with PCM cell array.

To implement the time window overlap method in a hardware experiment, we adapted a previously published field‐programmable gate array (FPGA)‐equipped SNN chip in **Figure** **3**a.^[^ ^24^, ^25^, ^37^ ^]^ The FPGA system is built with a commercially available FPGA board: Xilinx ZC706, a custom‐designed daughter board, a packaged module, and a host PC. An SNN chip is packaged into a ceramic pin grid array (PGA) module with wire bondings, then the packaged module is inserted into a PGA socket on the daughter board which is connected to the FPGA board through a board‐to‐board connector. There are DC/DC converters designed on the daughter board so that all necessary voltages can be supplied to the chip with some tunable voltage ranges. Instead of using the on‐board DC/DC converters, and external power supplies, a Keysight E3631A DC power supply in our case, can be also connected to the daughter board for easiness of control and remote accessibility. This chip features a 6T2R unit cell architecture based on PCM synaptic devices that store analog weights, complemented by a surrounding circuit that facilitates stochastic LIF neurons, allowing for energy‐efficient spiking through asynchronous and parallel operations. Furthermore, it includes a random walk circuit for stochastic neurons and essential SNN functions, such as refractory periods and leak mechanisms, with operational details elaborated in previous studies.^[^ ^24^, ^25^, ^37^ ^]^ These previous studies employed cells with 6T2R structures to implement functionalities such as spike‐timing‐dependent plasticity and backward LIF, which are not required in our method to solve the Max‐Cut problem. Therefore, synaptic devices with a reduced number of transistors, such as 2T2R and 1T1R structures, can be used for hardware demonstration. In addition, other emerging memories are allowed in synaptic devices.

For our hardware experiment on the weighted Max‐Cut problem, we initiated the process by mapping the problem onto a PCM cell array via weight transfer. Figure 3b illustrates the 5‐node Max‐Cut problem used in the hardware experiment. Figure 3c shows the corresponding weight matrix. Within our SNN chip, the total weight is represented by a signed synaptic weight obtained by pairing two half‐unit cells, which is calculated as *w*  =  *G*_p_  − *G*_m_. This approach enables the mapping of both negative weights (Equation (11)) and positive biases (Equation (12)) to the PCM cell array. By leveraging the gradual SET programming feature of the PCM, we program the cells with appropriate conductance by accessing the correct cell locations. The weight transfer is verified by measuring the final output spike through the integrate‐and‐fire mechanism, which is triggered only by the input spikes.

Following weight transfer, we verified the stochasticity of LIF neurons via random walk and analyzed the resulting firing rates. The firing rate is determined by tracking the output spikes generated when the random walk circuitry and leak function are activated for a specific period. Using this technique, the threshold voltage was adjusted and charted against the firing rate. Figure 3e shows the firing rates of five hidden neurons used in the hardware experiment, where the refractory period of the hidden neurons is 2.5 ms and the maximum firing rate is 400 Hz. The shape of a sweeping *V*_th_ shown in Figure 3e slightly differs from that shown in Figure S2c (Supporting Information, sweeping *u*_s_).

The Max‐Cut solving process was constructed using built‐in functions in SNN chip and FPGA configurations. The SNN chip features scan chain‐based I/O interfaces that facilitate simultaneous input and output of spikes across multiple neurons. Within this chip, access to the spikes is managed by reading or writing the scan latch values within the scan chain designated for each neuron index. This setup is programmed to deliver scan chain data directly to the FPGA register. Figure 3d illustrates the hardware implementation of the time window overlap method using both an SNN chip and an FPGA. For each neuron in the visible layer, PPD spike files are stored in the double‐data‐rate memory of the FPGA, and they are ready to be applied and allocated based on the neuron and time indices. The determination of neuron states in the Max‐Cut solving scenario relies on the output spikes from hidden neurons within a specified time window. The firing status is registered in the scan latch corresponding to the index of each neuron, after which the on‐chip scan chain data are periodically transferred and saved in the FPGA register at time intervals. At the end of each time window, the neuron firing index is evaluated via an OR operation among the registers that have been updated during the time window. The bias index, which indicates the bias neurons as “1,” is also incorporated into the neuron index mask via an OR operation. The final neuron index mask is then applied to the weight neurons in the visible layer using an AND operation, allowing for selective passage of external input spikes based on the neuron index. This procedure is systematically repeated throughout the Max‐Cut sampling phase. Figure 3f shows the outcome of addressing the Max‐Cut problem on our SNN chip equipped with a PCM cell array, demonstrating the ability of the experiment to achieve convergence toward a solution. In addition, the hardware results indicate the feasibility of solving COPs using the proposed sBM framework on hardware with hardware‐friendly approaches, OR operation for state update and random walk for stochasticity. Given that the SNN chip was not originally designed for the purpose of solving the Max‐Cut problem, we leveraged its existing functions and circuits, which led to insufficient memory to store the state at every time interval. Therefore, due to these storage limitations, only selective information from specific intervals is stored and depicted.

### 2.3 Additional Breakthroughs for Performance Improvement

Several additional breakthroughs are required to significantly aid in solving the Max‐Cut problem, as demonstrated by simulations and experiments. In this section, we propose additional methods that can be adjusted in the proposed method. The problems in this section were created by Rudy.^[^ ^35^ ^]^ Their weight conditions are identical to those in Figure 2c.

#### 2.3.1 Bias Split Methods for Steadier Membrane Potential

As stated previously, biases are assumed to be provided via spike trains, which differs from the ideal model for hardware brevity. In other words, Equation (5) is modified to Equation (16).

(16) $$u_{k}\left(t\right)=u_{\mathrm{rst}}+b_{k}\kappa \left(t-t_{b_{k}}^{s}\right)+\Sigma_{l\;=\;1}^{N}w_{\mathrm{kl}}\Sigma_{s\;=\;1}^{S}\kappa \left(t-t_{k}^{s}\right)$$

where $t_{b_{k}}^{s}$, $t_{k}^{s}$ denotes the spike timing of the input spike train generated by a specific stochastic process. We experimentally confirmed that the modified form does not significantly affect Max‐Cut solving when the number of nodes is small. However, as the number of nodes and weight density in the problem increase, we encounter a significant challenge: the amplitude of a single bias, which is the sum of all connected weights, increases. Thus, the discrepancy between the absolute value of an individual weight and a single bias weight is pronounced. This discrepancy amplifies the influence of bias spikes on the membrane potential, leading to deviations between the desired average firing rate ρ_D_(*u*_s_) in Equation S7 (Supporting Information), and the actual average firing rate obtained using real input spikes.

**Figure 4:**
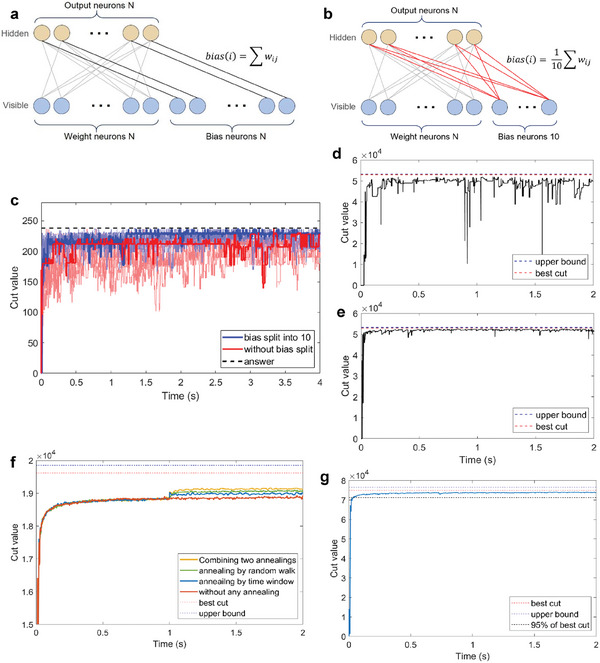
Additional breakthroughs to improve performance, bias split method and annealing methods. a) Example of the original network without bias split scheme. b) Example of network with bias split scheme into 10 neurons. c) Simulation results of 10 trials comparing the bias split method to the original method, solving 20‐node Max‐Cut problem with 30% weight density. Simulation results of solving 200‐node problem with 90% weight density with d) bias split into 10, e) bias split into 100. In (d–g), red lines and blue lines are the best cut values and upper bounds from BiqMac^[^ ^36^ ^]^ which provides the best cut value and upper bound value that is computed within a fixed time frame when the problems are large. f) Simulation results with various annealing strategies. The problem addressed is 200‐node 30% weight density, and bias split into 100 bias neurons is used. This figure indicates average cut values of 100 trials with each strategy. g) The average cut value of 100 samplings for 400‐node 30% weight density problem, using the bias split method into 200 bias nodes.

To address this issue, we introduce a bias weight splitting strategy that distributes bias weights across multiple bias neurons. This adjustment, by modifying the original scheme shown in **Figure** **4**a, involves setting the number of bias neurons equal to N and connecting all N bias neurons to each output neuron, an equal fraction of the total bias weight. This network configuration is exemplified in Figure 4b in which 10 bias neurons share the bias weight equally, contributing to a steadier membrane potential and approximating the average firing rate more closely with ρ_D_(*u*_s_).

Splitting the bias weight among a larger number of neurons yields a steadier membrane potential and reduces the variance in the firing rate. This is analogous to the situation presented in Figure S1b (Supporting Information), in which increasing the number of input nodes helps the system converge toward the average membrane potential. With sufficient burn‐in time, the membrane potential increment converges to the stationary potential for the given bias spikes. This situation can be likened to that of the ideal model, where biases shift the resting potential of the neurons. The proposed bias split method effectively improves the accuracy of the search process without the need for additional hardware components for biases. Figure 4c illustrates that the proposed method successfully identifies the exact solution for smaller‐scale problems and demonstrates that the bias split approach leads to faster convergence to higher values. Figure 4d,e presents the simulation results for a 200‐node Max‐Cut problem with a 90% weight density, employing the time window overlap method and bias split scheme with 10 and 100 bias neurons under the same simulation conditions. These simulations reveal that NP‐hard problems such as Max‐Cut, whose complexity increases with increasing node and density, benefit from the bias split approach, converging toward the upper bounds. Figure 4d shows some fluctuations due to the dense and complex energy landscape, whereas Figure 4e demonstrates smoother convergence with a larger number of split bias neurons.

Our results suggest that this scheme can achieve rapid convergence near‐optimal solutions even for larger Max‐Cut problems and may yield improved outcomes when combined with annealing techniques, which will be discussed in detail later in this study. Notably, in scenarios involving the 200‐node problem and even 400‐node problem in Figure 4g, the proposed method exhibits robust performance with a small number of split bias neurons, potentially reducing the hardware footprint and operational power requirements. This approach underscores that, in NP‐hard problems, effective management of bias weights and neuron configurations can maintain high‐performance levels, thereby facilitating rapid convergence even in large and complex problem landscapes without additional neurons or cell arrays. In addition, for abnormally large weights, weight split can be considered in the same context.

#### 2.3.2 Simulated Annealing Methods for Improved Convergence

Our method is based on BMs, which enables us to employ simulated annealing strategies to improve convergence in heuristic approaches based on energy‐based systems. Simulated annealing, which is characterized by a gradual reduction in temperature during the search process, helps reduce the randomness in searching, ultimately converging the system toward an optimal solution.^[^ ^38^ ^]^ We introduce two hardware‐friendly annealing strategies within the sBM framework: “annealing by random walk” and “annealing by time window.” Each method leverages different aspects of the system dynamics to reduce randomness and narrow the search space over time.

##### Annealing by Random Walk

This approach modulates the temperature of the system by adjusting the random walk step size of Diffusive LIF neurons. By decreasing the step size while keeping ρ_D_(*u*_rst_) constant, the system temperature can be effectively reduced. In Figure 4f, the impact of annealing by random walk is illustrated, showing improved convergence after a predefined temperature decrease of 8% at 1 s. A simple stepwise reduction in the random walk step size is an effective compromise. In addition, annealing by random walk can be efficiently implemented on hardware because the random walk step size can be changed by simply adjusting the bias voltages to the random walk circuit.^[^ ^25^ ^]^

##### Annealing by Time Window

This method extends the length of the time window and modifies the threshold for the number of spikes determining neuron states (0 or 1) during sampling, aligning more closely with the desired firing rate (ρ_D_(*u*_s_)). The approach of annealing by time window allows for the realization of annealing benefits without modifying parameters such as the random walk step size. By extending the time window length with a factor of *k*, the state of a neuron is determined as 1 if it generates at least *k* outputs during the extended period. As shown in Figure S7 (Supporting Information), elongating the time window decreases the variance between the actual firing rates of neurons and ρ_D_(*u*_s_), thereby improving search accuracy. Moreover, the variance in counts decreases as the duration of the counting window increases, as demonstrated by the PPD.^[^ ^39^ ^]^ However, although longer time windows can improve convergence accuracy, there is a trade‐off with the delay in reflecting output spikes to state updates. In Figure S5 (Supporting Information), when the bias is split among a sufficient or large number of neurons, the approach of annealing by time window adjustments appears to be effective by demonstrating that starting with a shorter time window and transitioning to a longer time window provides a balanced approach for achieving rapid initial progress, followed by higher convergence. Figure 4f shows the effectiveness of annealing by time window, exhibiting improved performance after 1 s. In Figure 4f, the threshold for the number of spikes determining the neuron state changes after 1 s and requires at least three outputs within a duration that is triple the original time window (2τ_ref_).

With these two annealing methods, narrower randomness during searching improves the sampling performance. Furthermore, the simultaneous use of both annealing methods further enhances convergence. These methods can be effectively used to solve COPs based on the spiking system, along with the bias split method.

#### 2.3.3 Flexible Annealing Considering Unstable Neurons

In annealing strategies, each annealing condition has distinct convergence rates and levels. It is advisable to start the process with settings that facilitate rapid progression and subsequently transition to conditions that improve convergence. The number of unstable neurons can serve as an indicator of the optimal time to modify the annealing conditions. When the exact solution to the Max‐Cut problem remains unknown, the cut value alone may not provide a reliable criterion for changing the annealing conditions. Despite the difference between the number of unstable neurons and the cut value, maintaining a smaller number of unstable neurons tends to result in a higher convergence level, both of which are rooted in the energy‐based function. Therefore, the time for changing the annealing conditions can be strategically determined by monitoring the number of unstable neurons during the sampling process, thereby offering a more dynamic and flexible annealing schedule than by adhering to a fixed annealing schedule. This flexible annealing schedule enables faster convergence and better performance.

#### 2.3.4 Circuit Ideas for Evaluating Convergence

To effectively solve the Max‐Cut problem, evaluating the degree of convergence by monitoring the trend of cut values or the number of unstable neurons can play a critical role. These assessments aid in fine‐tuning hyperparameters, devising strategic annealing schedules, and applying flexible annealing schedules. During our simulations and experiments, the cut values are calculated and the unstable neurons are counted using software every time, these could be inefficient. Implementing these evaluations on neuromorphic hardware will also improve efficiency. We develop innovative circuit designs using PCM cell arrays to carry out these evaluations directly on the hardware, thereby removing the need for external software or computational tools.

##### Cut Value Calculation Circuit

**Figure 5:**
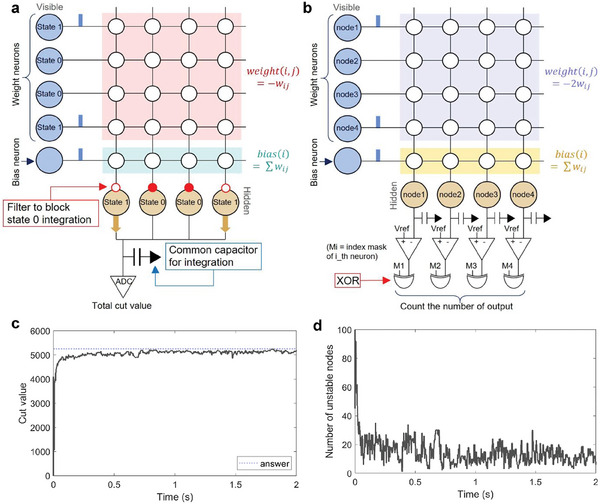
Circuit designs to evaluate the convergence of sampling. a) Cut value calculation circuit idea, b) unstable neuron count circuit idea using PCM cell array. c) Example of cut value graph solving 100‐node 30% weight density Max‐Cut problem with bias split into 100, d) example of the number of unstable neurons corresponding to (c).

As shown in **Figure** **5**a, this circuit concept is engineered to accurately calculate the cut value. The *weight*(*i*,  *j*) and *bias*(*i*) values in an array, as in Figure 5a, are based on the equation $f\left(x\right)=\sum_{i=1}^{N}\left(\sum_{j\;=1}^{N}w_{\mathrm{ij}}\right)\cdot x_{i}+\sum_{i}\sum_{j}\left(-w_{\mathrm{ij}}\right)\cdot x_{i}\cdot x_{j}$ derived from rearranging the cost function of Max‐Cut (Equation (9)). Unlike earlier neuron operations, this circuit design excludes the leak and random walk functions and focuses solely on integration to precisely determine the cut value without requiring a probabilistic distribution. The visible layer neurons represent neuron states and bias, whereas the hidden layer outputs are integrated into a shared capacitor that aggregates the total cut value. Only one bias neuron and synchronous firings from neurons are adequate. The hidden layer integrates charge or discharge only from neurons in state 1 using filters, such as transistors or data selectors, to prevent contributions from neurons in state 0 to the common capacitor. The accumulated membrane potential, which was read using an analog‐to‐digital converter, reflects the cut value.

##### Unstable Neuron Count Circuit

As shown in Figure 5b, this circuit facilitates the counting of unstable neurons, as defined by the conditions in Equation (15). Mirroring the cut value circuit in simplicity eliminates the need for a random walk or leak functions; only one bias neuron and synchronous firings from neurons are adequate. However, it features individual capacitors and comparators for neurons in the hidden layer to assess the weighted sum against each neuron state. The *weight*(*i*,  *j*) and *bias*(*i*) values in the array, as shown in Figure 5b, are grounded in Equation (13), and the reference voltage of the comparator (*V*_ref_), acting as the voltage at which the weighted sum equals zero, enables the comparison of the weighted sum with 0. The XOR operation between the comparator outputs and neuron states identifies unstable neurons; from the XOR results, the number of unstable neurons can be obtained.

These circuit designs provide a parallel and energy‐efficient solution for calculating cut values and counting unstable neurons in an SNN system. Once a weight‐transferred array has been configured, these circuits allow for iterative reuse of the array by merely adjusting neuron states, which demonstrates their versatility. Furthermore, these concepts are adaptable to other emerging memory technologies, broadening their application potential in neuromorphic computing.

## 3 Conclusion

In this study, we introduce an innovative approach for solving the Max‐Cut problem with an sBM framework using stochastic LIF neurons in conjunction with SNN‐based neuromorphic hardware consisting of a PCM synaptic array. The proposed time window overlap method proved effective in simulations, and we successfully mapped and solved the Max‐Cut problem on a specialized SNN‐based neuromorphic chip. Considering the properties of SNN, we proposed additional methods that demonstrated improved convergence toward solutions to large or densely connected problems. Furthermore, two circuit designs to evaluate sampling convergence were introduced, suggesting a new paradigm of utilizing a neuromorphic cell array.

This study is, to our knowledge, the first study to solve the Max‐Cut problem with an SNN neuromorphic hardware chip. By exploiting the asynchronous spiking properties and the temporal dimensions inherent in spike‐based systems, the proposed methods demonstrate the capacity of sBM systems for effectively solving COPs, highlighting the feasibility of hardware implementation. Our study advances neuromorphic computing applications and paves the way for further exploration of COPs, marking a significant step forward in this field.

## Conflict of Interest

The authors declare no conflict of interest.

## Supporting information



Supporting Information

## Data Availability

The data that support the findings of this study are available from the corresponding author upon reasonable request.
