# Supplementary material for: Solving Max‐Cut Problem Using Spiking Boltzmann Machine Based on Neuromorphic Hardware with Phase Change Memory
Source: Adv Sci (Weinh). 2024 Oct 23;11(46):2406433. doi: 10.1002/advs.202406433 (PMC11633485; doi:10.1002/advs.202406433)
Supplement: Supplementary file 1 — Supporting Information [file ADVS-11-2406433-s001.docx]

Supporting Information

Solving Max-Cut Problem using Spiking Boltzmann Machine based on Neuromorphic Hardware with Phase Change Memory

Yu Gyeong Kang, Masatoshi Ishii, Jaeweon Park, Uicheol Shin, Suyeon Jang, Seongwon Yoon, Mingi Kim, Atsuya Okazaki, Megumi Ito, Akiyo Nomura, Kohji Hosokawa, Matthew BrightSky*, and Sangbum Kim*

**S1 Calibration method for three suggested models**

To ensure an equitable evaluation of the three models, the calibration involves adjusting the exponential PSP model's scaling factor, using parameters such as $\tau_{leak}, \tau_{ref}$, and determining the parameter $T$ in the escape noise model based on $v_{up}$, $v_{dn}$ from the Diffusive noise model.

The calibration of the exponential PSP model's scaling factor is based on the defined stationary membrane potential. Consider a neuron, indexed as $k$, that receives spikes from all other neurons, where each neuron's spike train follows a specific probabilistic process. Then the membrane potential of neuron $k$, $u_{k}$, changes as the sum of the PSP kernel $\kappa$ applied to all received spikes as in Equation (5).

We postulate that a sufficiently large number of neurons exists and highly frequent and smaller PSPs are generated. This leads to the average membrane potential across all possible spike trains, denoted as $<u_{k}\left( t \right)>$, closely approximating the actual membrane potential $u_{k}\left( t \right)$. This relationship is substantiated by the data presented in Figure S1, which illustrates the predicted $<u_{k}\left( t \right)>$ alongside randomly selected instances of $u_{k}\left( t \right)$ for networks comprising 10, 100, and 1000 nodes.

When spike generation adheres to a specific stochastic process, such as the Poisson distribution, the mean membrane potential $<u_{k}\left( t \right)>$ stabilizes over time, converging to a consistent value as $t\to\infty$, which is referred to as the stationary membrane potential $u_{s}$. We assume that our Max-cut solving method employs the Poisson process with dead-time (PPD) as a spike generation mechanism. In this process, spikes are generated with a rate parameter λ, and each spike is succeeded by a refractory period $\tau_{ref}$ precluding subsequent firings. This deviation from a pure Poisson process introduces complexities in calculating the exact average membrane potential. To handle this, we approximate the superposition of given processes as the Poisson process with the rate parameter $\frac{N}{\frac{1}{\lambda}+\tau_{ref}}$. See Supporting Information Section 2 for detailed explanation.

For this Poisson process, $<u_{k}\left( t \right)>- u_{rst}-b_{k}$ is exactly given as a product of the average PSP contribution to membrane potential $\frac{\Sigma_{l=1}^{N}w_{kl}}{N}<\kappa\left( t-s \right)>$ and the average spike firing rate $\frac{N}{\frac{1}{\lambda}+\tau_{ref}}$. Then $<u_{k}\left( t \right)>$ and $u_{s}$ is calculated:

$<u_{k}\left( t \right)> = u_{rst}+b_{k}+ A_{r}\cdot\Sigma_{l=1}^{N}w_{kl}\cdot\frac{1}{\frac{1}{\lambda}+\tau_{ref}}\cdot\min\left( t, \tau_{ref} \right) for rectangular PSP$ (S1)

$<u_{k}\left( t \right)> = u_{rst}+b_{k}+ A_{e}\cdot\Sigma_{l=1}^{N}w_{kl}\cdot\frac{1}{\frac{1}{\lambda}+\tau_{ref}}\cdot\tau_{leak}\cdot\left( 1-e^{\frac{t}{\tau_{leak}}} \right) for exponential PSPs$ (S2)

and

$u_{s}=u_{rst}+b_{k}+ A_{r}\cdot\Sigma_{l=1}^{N}w_{kl}\cdot\frac{\tau_{ref}}{\frac{1}{\lambda}+\tau_{ref}} for rectangular PSPs$ (S3)

$u_{s} = u_{rst}+b_{k}+ A_{e}\cdot\Sigma_{l=1}^{N}w_{kl}\cdot\frac{\tau_{leak}}{\frac{1}{\lambda}+\tau_{ref}} for exponential PSPs$ (S4)

By setting stationary membrane potential for two PSP cases the same, we used the scaling factor of $\frac{A_{e}}{A_{r}}=\frac{\tau_{ref}}{\tau_{leak}}$.

To calibrate the parameter $T$ in the escape noise model based on the parameters $v_{up}$ and $v_{dn}$ in Diffusive noise model, we derive the equation for average firing rate $\rho$ in terms of the stationary membrane potential, i.e. the inverse of the sum of the refractory time and the average time spent for the first spike right after the refractory period. For the three models, the equations are given below, see Supporting Information Section 3 for derivation:

$\rho_{A}\left( u_{s} \right)=\left( \tau_{ref}\left( 1+e^{-\frac{u_{s}}{\tau_{ref}}} \right) \right)^{-1}for Abstract neuron$ (S5)

$\rho_{E}\left( u_{s} \right)=\left( \tau_{ref}+\int_{0}^{\infty} \frac{x}{\tau_{ref}}e^{\frac{u_{s}-u_{rst}}{T_{E}}\cdot\left( 1-e^{-\frac{x}{\tau_{leak}}} \right)+\frac{\tau_{leak}}{\tau_{ref}}\cdot e^{\frac{u_{s}-u_{rst}}{T_{E}}}\cdot\left( Ei\left( -\frac{u_{s}-u_{rst}}{T_{E}}e^{-\frac{x}{\tau_{leak}}} \right)-Ei\left( -\frac{u_{s}-u_{rst}}{T_{E}} \right) \right)}dx \right)^{-1}$

$for Escaping LIF neuron$ (S6)

where $Ei(\cdot)$is exponential integral function,

$\rho_{D}\left( u_{s} \right)\cong\left( {\tau_{ref}+\tau}_{leak}\sqrt{\pi}\int_{\frac{u_{rst}-u_{s}-m}{\sigma}}^{\frac{\theta-u_{s}-m}{\sigma}} e^{x^{2}}\left( 1+Erf\left( x \right) \right)dx \right)^{-1} for Diffusive LIF neuron$ (S7)

where $m$= $\frac{\tau_{leak}}{{2t}_{step}}$ $(v_{up}{-v}_{dn}$), and $\sigma$= $\frac{\tau_{leak}^{0.5}}{2t_{step}^{0.5}}$ $(v_{up}{+v}_{dn}$), $Erf(\cdot)$ is error function.

Figure S2, depicts the correlation between predicted and simulated stationary membrane potentials for the three models, ensuring the correctness of derived equations.

The parameter $T$ should be set such that the average firing rates of escape neurons and diffusive neurons are similar. For this, we calibrate m and $\sigma$ such that $\rho_{D}\left( u_{s} \right)$ = $\frac{1}{2\tau_{ref}}$ to obtain proper $v_{up}$ and $v_{dn}$, as $\rho_{A}\left( u_{rst} \right)=\frac{1}{2\tau_{ref}}$, $\rho_{E}\left( u_{rst} \right)=\frac{1}{2\tau_{ref}}$ regardless of $T$ by setting $u_{s}\to u_{rst}$ in Equation (S5) and (S6). Also, since the membrane potential of neurons stays near $u_{rst}$ the most of time, we set $\rho'\left( u_{rst} \right)$ for the three neuron models the same. As

${\frac{{d\rho}_{A}\left( u_{s} \right)}{du_{s}}|}_{\rho_{A}\left( u_{s} \right)=\frac{1}{2\tau_{ref}}}=\frac{1}{4T_{A}\tau_{ref}}$ , (S8)

${\frac{{d\rho}_{E}\left( u_{s} \right)}{du_{s}}|}_{\rho_{E}\left( u_{s} \right)=\frac{1}{2\tau_{ref}}}=\frac{1}{4T_{E}(\tau_{ref}+\tau_{leak})}$ , (S9)

and

${\frac{{d\rho}_{D}\left( u_{s} \right)}{du_{s}}|}_{\rho_{D}\left( u_{s} \right)=\frac{1}{2\tau_{ref}}}=\frac{\sqrt{\pi}\tau_{leak}}{4t_{ref}^{2}\sigma}(e^{\left( \frac{\theta-u_{rst}-m}{\sigma} \right)^{2}}(1+erf\left( \frac{\theta-u_{rst}-m}{\sigma} \right)) -e^{\left( \frac{-m}{\sigma} \right)^{2}}(1+erf\left( \frac{-m}{\sigma} \right))$ , (S10)

the parameter $T$ is calculated by setting Equation (S8), (S9) and (S10) the same.

$T_{E}=\frac{\tau_{ref}^{2}\sigma}{\sqrt{\pi}\tau_{leak}(\tau_{ref}+\tau_{leak})}(e^{\left( \frac{\theta-u_{rst}-m}{\sigma} \right)^{2}}(1+\left( \frac{\theta-u_{rst}-m}{\sigma} \right)) -e^{\left( \frac{-m}{\sigma} \right)^{2}}(1+\left( \frac{-m}{\sigma} \right)))$ , (S11)

$T_{A}=T_{E}\cdot\frac{t_{ref}+t_{leak}}{t_{ref}}$ (S12)

The average firing rate of the Escaping LIF neuron model and that of the Diffusive neuron model appears to be analogous when calibration is done as shown in Figure S3. We further tried the mean-squared error minimization but the temperature values barely changed.

**S2 Stationary potential**

The stationary potential is defined as the converging value of the averaged membrane potential of the post-neuron. To calculate the stationary potential in our Max-Cut solving scheme, we assumed that the post-neuron is connected to $N_{pre}$ pre-neurons and the spike train from each pre-neuron follows the Poisson process with dead time (PPD) of rate parameter $\lambda$ independently and identically. Dead time is equal to refractory period. Weights connecting the post-neuron and pre-neurons are sampled from normal distribution and normalized to have certain mean and variance. The size and timing of PSPs from pre-neurons is set by the determined weights and spike train, and PSPs are accumulated to the membrane potential of the post-neuron. We simulated the stationary potential with $N_{pre}$of 10, 100, 1000 and weights were set to $N(\frac{1}{N_{pre}},\frac{1}{N_{pre}})$. The simulation results are shown in Figure S1.

To calculate stationary potential mathematically, we approximate the superposition of PPDs to pure Poisson process. Although the coefficient of variation of the superposition is not precisely 1, it converges to 1 as $N_{pre}$ increases. And the serial coefficient caused by non-renewal process is negligibly small. We refer to previous study^[1]^ for the assumptions and equations derived. The approximated Poisson process have rate factor of $\frac{N_{pre}}{\frac{1}{\lambda}+\tau_{ref}}$.

For Poisson process, we can calculate averaged membrane potential and variance exactly. The membrane potential with given PSP $\kappa(t)$ is:

$u_{post}(t)= u_{rst} + b_{post} \kappa(t-t_{b_{post}}^{s} )+ \Sigma_{l=1}^{N} W_{l} \Sigma_{s=1}^{S} \kappa(t-t_{l}^{s} )$

$= u_{rst} + \Sigma_{s:t_{s}<t} A_{i}\kappa(t-t^{s} )$

where $A_{i}$ denotes the random variable uniformly sampled from weights and bias, $t^{s}$ denotes the superposed spike train. The biases are expressed as spike trains with firing rates the same as input spike trains, as in our hardware bias implementation.

Then averaged membrane potential is:

${<u}_{post}\left( t \right)> = E\left[ u_{rst} + \Sigma_{s:t_{s}<t} A_{i}\kappa\left( t-t^{s} \right) \right]$

$= u_{rst} + \Sigma_{s:t_{s}<t} E\left[ A_{i} \right]\cdot\kappa\left( t-t^{s} \right)$

$= u_{rst} + \frac{N}{\frac{1}{\lambda}+t_{ref}}\int_{0}^{t} E\left[ A_{i} \right]\cdot\kappa\left( t-s \right)ds$

$= u_{rst} + \frac{N}{\frac{1}{\lambda}+t_{ref}}\cdot\frac{b_{post}+\Sigma_{l=1}^{N}w_{l}}{N}\cdot\int_{0}^{t} \kappa\left( t-s \right)ds$

$=u_{rst} +(b_{post} + \Sigma_{l=1}^{N}w_{l})\cdot\frac{1}{\frac{1}{\lambda}+t_{ref}}\cdot\int_{0}^{t} \kappa\left( t-s \right)ds$

For rectangular PSPs with $\kappa\left( t \right)=\Theta\left( t-t^{s} \right)\Theta\left( t^{s}-t+\tau_{ref} \right)$,

${<u}_{post}\left( t \right)>=u_{rst} +(b_{post}+ \Sigma_{l=1}^{N}w_{l})\cdot\frac{1}{\frac{1}{\lambda}+t_{ref}}\cdot\int_{0}^{t} \Theta\left( t-s-t^{s} \right)\Theta\left( t^{s}-t+s+\tau_{ref} \right)ds$

$= u_{rst}+(b_{post}+ \Sigma_{l=1}^{N}w_{l})\cdot\frac{1}{\frac{1}{\lambda}+\tau_{ref}}\cdot min(t, \tau_{ref})$

$u_{s}=u_{rst}+(b_{post}+ \Sigma_{l=1}^{N}w_{l})\cdot\frac{\tau_{ref}}{\frac{1}{\lambda}+\tau_{ref}}$

For exponential PSPs with $\kappa\left( t \right)=\frac{\tau_{ref}}{\tau_{leak}}\cdot e^{-\frac{s}{\tau_{leak}}}$,

${<u}_{post}\left( t \right)>=u_{rst} +(b_{post}+ \Sigma_{l=1}^{N}w_{l})\cdot\frac{1}{\frac{1}{\lambda}+t_{ref}}\cdot\int_{0}^{t} \frac{\tau_{ref}}{\tau_{leak}}\cdot e^{-\frac{s}{\tau_{leak}}}ds$

$= u_{rst}+(b_{post}+ \Sigma_{l=1}^{N}w_{l})\cdot\frac{\tau_{ref}}{\frac{1}{\lambda}+\tau_{ref}}\cdot\left( 1-e^{\frac{t}{\tau_{leak}}} \right)$

$u_{s} = u_{rst}+(b_{post}+ \Sigma_{l=1}^{N}w_{l})\cdot\frac{\tau_{ref}}{\frac{1}{\lambda}+\tau_{ref}}$

By inserting parameters of Figure S1 in Table S1, we obtain $u_{s}=0.833\ldots$ for both rectangular PSPs and exponential PSPs.

**S3 Average firing rate**

The average firing rate is defined as average spike count per unit time with given stationary input. To derive the average firing rate, it is assumed that a huge number of neurons are available such that the impact of a single spike is negligible and the synaptic input of neurons is only described by its average, i.e., mean-field theory is applied.

We first derive the average firing rate for the Abstract neuron and the Escaping LIF neuron.

The survivor function $S(t)$is the probability of the neuron not fired in time interval $t$after previous firing. It is set to $S(0)=1$ and $S(\infty)=0$. Then the instantaneous rate of firing $\rho\left( t \right)=-\frac{\frac{d}{dt}S(t)}{S(t)}$. By integrating the differential equation yields:

$$S\left( t \right)=e^{-\int_{0}^{t} \rho\left( t' \right)dt'}$$

With this, the interspike interval distribution is $ISI\left( t \right)=\rho\left( t \right)\cdot S\left( t \right)=\rho\left( t \right)\cdot e^{-\int_{0}^{t} \rho\left( t' \right)dt'}$.

The average firing rate is equal to the inverse of the sum of the refractory time and the average inter-spike interval.

$$\rho_{avg}\left( u_{s} \right)=\frac{1}{\tau_{ref}+\int_{0}^{\infty} t\cdot\rho\left( t \right)\cdot e^{-\int_{0}^{t} \rho\left( t^{'};u_{s} \right)dt'}dt}$$

For the Abstract neuron and the Escaping LIF neuron, $\rho\left( t \right)=\tau_{ref}^{-1}\cdot exp(\frac{u\left( t \right)-u_{rst}}{T})$ as in Equation (2). We assume $u(t)$ as averaged membrane potential $<u\left( t \right)>$. $u\left( t \right)=u_{s}$ for the Abstract neuron and $u\left( t \right)=u_{rst}+u_{s}\cdot\left( 1-e^{\frac{t}{\tau_{leak}}} \right)$ for the Escaping LIF neuron. By substituting we obtain:

$$\rho_{A}\left( u_{s} \right)=\left( \tau_{ref}\left( 1+e^{-\frac{u_{s}}{\tau_{ref}}} \right) \right)^{-1}for Abstract neuron$$

$$\rho_{E}\left( u_{s} \right)=\left( \tau_{ref}+\int_{0}^{\infty} \frac{x}{\tau_{ref}}e^{\frac{u_{s}-u_{rst}}{T_{E}}\cdot\left( 1-e^{-\frac{x}{\tau_{leak}}} \right)+\frac{\tau_{leak}}{\tau_{ref}}\cdot e^{\frac{u_{s}-u_{rst}}{T_{E}}}\cdot\left( Ei\left( -\frac{u_{s}-u_{rst}}{T_{E}}e^{-\frac{x}{\tau_{leak}}} \right)-Ei\left( -\frac{u_{s}-u_{rst}}{T_{E}} \right) \right)}dx \right)^{-1}$$

$$for Escaping LIF neuron$$

For the Diffusive LIF neuron with random walk, we derive the average firing rate by modifying the derivation in previous study.^[2]^ The neuron membrane potential dynamics can be solved by Equation (6):

$\tau_{leak}\frac{d}{dt}u_{k}= -(u_{k} - u_{rst}) + I\left( t \right) + \xi, u_{k}\left( t \right)\in\left( -\infty,\theta\right)$

With this the membrane potential diffuses with random walk and linear drift is given by leakage. By Donsker’s theorem,^[3]^ for sufficiently small random walk size, we can approximate random walk with the Brownian motion. With this approximation the dynamics of the membrane potential given in Equation (6) is described by the Ornstein-Uhlenbeck (O-U) process. The probability density of membrane potential at given time $t$ and potential $u$ by the dynamics of O-U process can be evaluated using Fokker-Planck equation:

$$\frac{\partial P\left( u^{0}, t^{0} \right)}{\partial t} =\frac{\partial}{\partial u}\left[ \frac{\left( u - u_{s}-m \right)}{\tau_{leak}} \cdot P\left( u^{0}, t^{0} \right) \right]+\frac{\sigma^{2}}{2\tau_{leak}}\cdot\frac{\partial^{2}P\left( u^{0}, t^{0} \right)}{\partial u^{2}}$$

where $P(\cdot)$ is the probability density function $m$= $\frac{\tau_{leak}}{{2t}_{step}}$ $(v_{up}{-v}_{dn}$), and $\sigma$= $\frac{\tau_{leak}^{0.5}}{2t_{step}^{0.5}}$ $(v_{up}{+v}_{dn}$). With boundary conditions:

$P(\theta,t^{0}|u^{0},t^{0})=0 and$ $\frac{\partial}{\partial u}P(\theta,t^{0}|u^{0}, t^{0})=-\frac{2\rho\cdot\tau_{leak}}{\sigma^{2}}$

${lim}_{u\to-\infty}P(u,t^{0}|u^{0}, t^{0})=0$ and ${lim}_{u\to-\infty}u\cdot P(u,t^{0}|u^{0}, t^{0})=0$.

Finding the solution for the steady-state distribution for this Fokker-Planck equation with boundary conditions given by threshold voltage yields the average firing rate:

$\rho_{D}\left( u_{s} \right)\cong\left( {\tau_{ref}+\tau}_{leak}\sqrt{\pi}\int_{\frac{u_{rst}-u_{s}-m}{\sigma}}^{\frac{\theta-u_{s}-m}{\sigma}} e^{x^{2}}\left( 1+Erf\left( x \right) \right)dx \right)^{-1} for Diffusive LIF neuron$

**References**

[1] M. Deger, M. Helias, C. Boucsein, S. Rotter, *J. Comput. Neurosci.* **2012**, *32*, 443.

[2] A. Renart, N. Brunel, X.-J. Wang, *Comput. Neurosci. a Compr. Approach* **2003**, DOI 10.1201/9780203494462.ch15.

[3] M. D. Donsker, *An Invariance Principle for Certain Probability Limit Theorems*, **1951**.

| **parameters** | **symbol** | **used values** | **corresponding simulations** | **notes** |
| --- | --- | --- | --- | --- |
| Simulation time |  | 0.020 s | Figure S1 |  |
|  |  | 1 s | Figure S2 |  |
|  |  | 110 s | Figure 1 |  |
|  |  | 4 s | Figure 2c, 4c, S5c&f |  |
|  |  | 2 s | Figure 4d-g, 5c&d, S4, S5a&b&d&e, S6, S8 |  |
|  |  | 0.1 s | Figure S7a&b |  |
|  |  | 0.2 s | Figure S7c&d |  |
|  |  | 0.3 s | Figure S7e&f |  |
|  |  | 0.4 s | Figure S7g&h |  |
| Simulation step time |  | ${10}^{-6}$ s | All figures |  |
| Random walk injection time | $t_{step}$ | ${10}^{-5}$ s | All figures |  |
| Burn-in time |  | 10 s | Figure 1 |  |
| Number of neurons | $N$ | 10, 100, 1000 | Figure S1a&b |  |
|  |  | 10 | Figure S7a&c&e&g |  |
|  |  | 100 | Figure S1c&d, S2, S7b&d&f&h |  |
|  |  | 30 | Figure 1 |  |
| Number of problem nodes |  | 100 | Figure 2c, 5c&d |  |
|  |  | 20 | Figure 4c |  |
|  |  | 200 | Figure 4d-f, Figure S4, S5, S6 |  |
|  |  | 400 | Figure 4g, Figure S8 |  |
| Weights | $w$ | N(0.1, 0.1)  N(0.01, 0.01)  N(0.001, 0.001) | Figure S1a&b | For 10-node, 100-node, 1000-node case each |
|  |  | N(0.01, 0.01) | Figure S1c&d |  |
|  |  | N(-, 0.03) | Figure S2 | Mean weights were modified for each data point |
|  |  | N(0, 0.03) | Figure 1 |  |
|  |  | N(-, 0.1) | Figure S7a&c&e&g |  |
|  |  | N(-, 0.01) | Figure S7b&d&f&h |  |
|  |  | - | Figure 2c, 4c-g, 5c&d, S4, S5, S6, S8 | Weights of the Max-Cut problem were used. |
| Biases | $b$ | 0 | Figure S1, S2 |  |
|  |  | N(-1.5, 0.75) | Figure 1 |  |
|  |  | - | Figure 2c, 4c-g, 5c&d, S4, S5, S6, S8 | Biases of the Max-Cut problem were used. |
| Refractory time | $\tau_{ref}$ | 0.004 s | All figures |  |
| Leakage time constant | $\tau_{leak}$ | 0.01 s | All figures |  |
| Rate factor of Poisson process when not in refractory period | $\lambda$ | 1250 Hz | All figures |  |
| Temperature of the Abstract neuron model | $T_{A}$ | 2.699 | Figure 2c |  |
| Temperature of the Escaping LIF neuron model | $T_{E}$ | 0.7710 | Figure 2c |  |
| Random walk voltage of the Diffusive LIF model | $v_{up}$/$v_{dn}$ | 0.04 / 0.0345 | Figure 1, 2c, 4c-g, 5c&d, S4, S5, S6, S7, S8 | 0.01 / 0.041 for annealing by random walk in Figure 4f, S6 |
| Firing threshold of the Diffusive LIF model | $\theta$ | 1 | All figures |  |
| Reset potential | $u_{rst}$ | 0 | All figures |  |

**Table S1.** List of parameters for the simulations


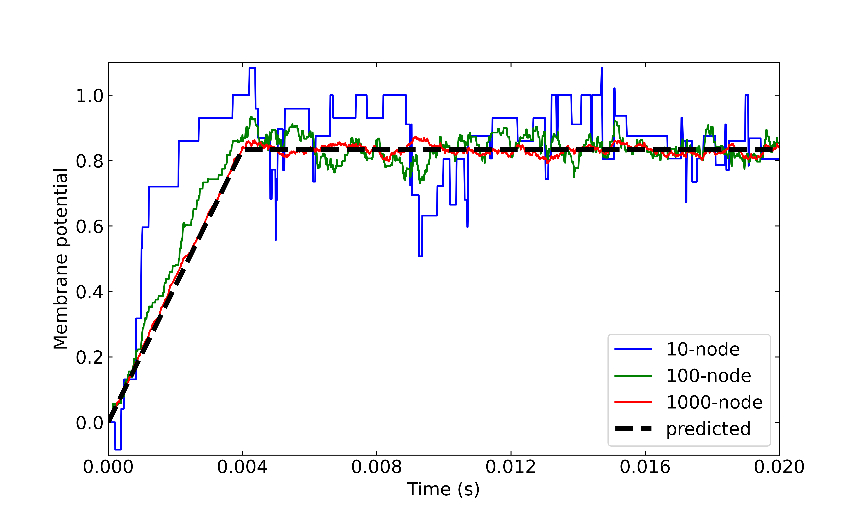


**a**


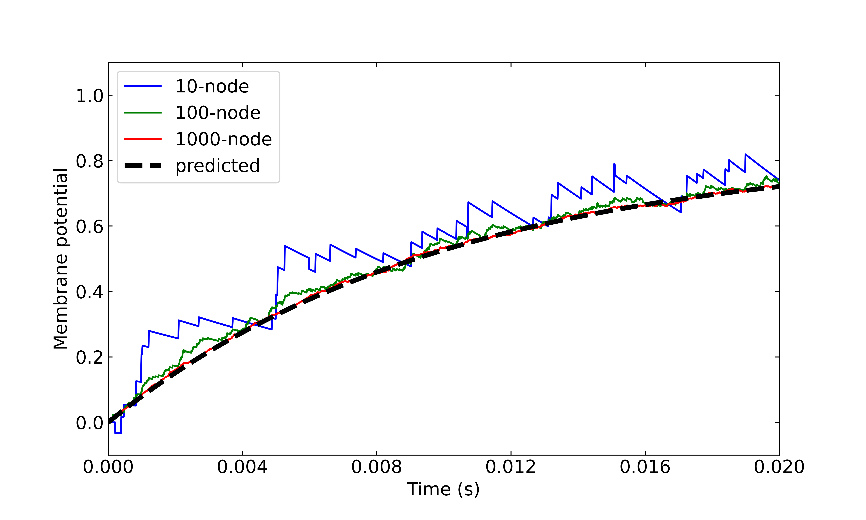


**b**


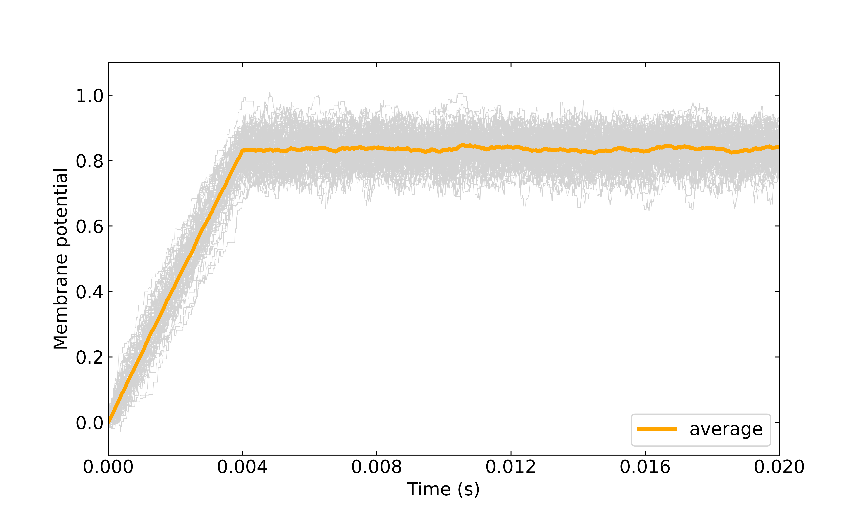


**c**


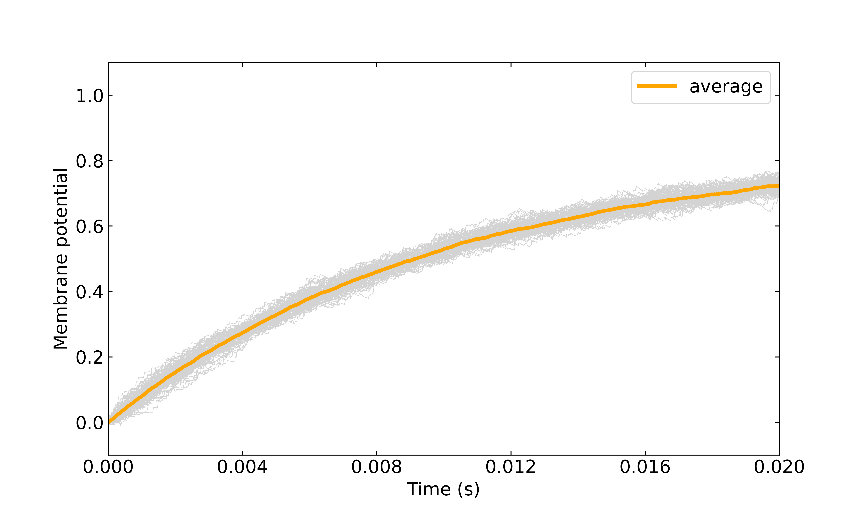


**d**

**Figure S1.** Predicted $<u_{k}\left( t \right)>$, randomly selected $u_{k}\left( t \right)$ of 10/100/1000-node case (a) rectangular PSP, (b) exponential PSP. Numerical simulation of $<u_{k}\left( t \right)>$, averaged over 100 numerical simulations of $u_{k}\left( t \right)$, (c) rectangular PSP, (d) exponential PSP. The averaged membrane potential converges to ~0.83, and predicted membrane potential with $\lambda$ =1250 Hz, $\tau_{ref}$ = 0.004 s, weighted sum = 1 is ⅚.


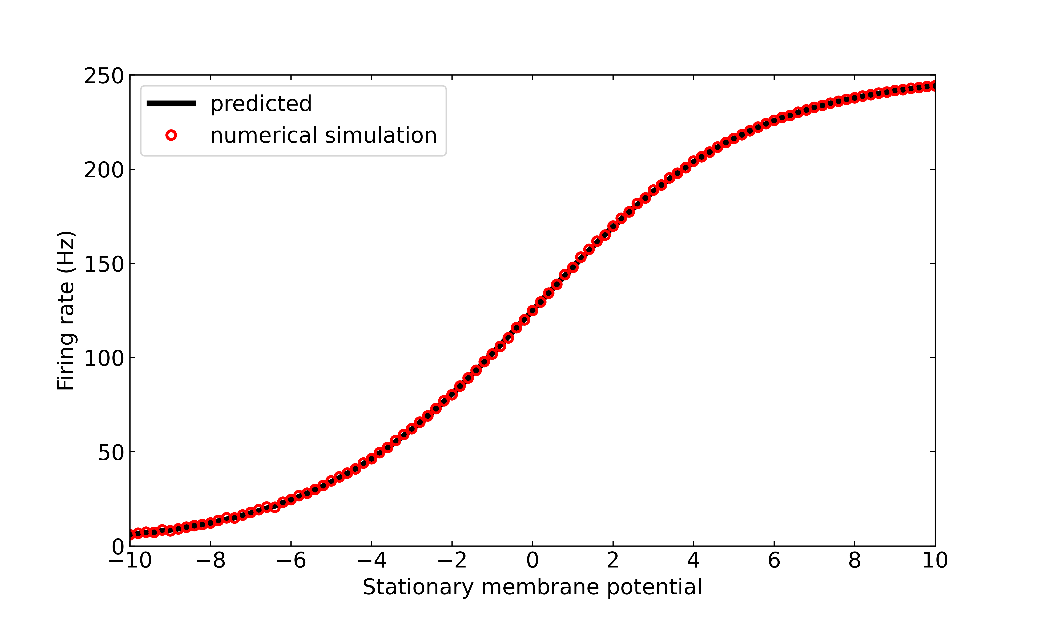


**b**

**a**


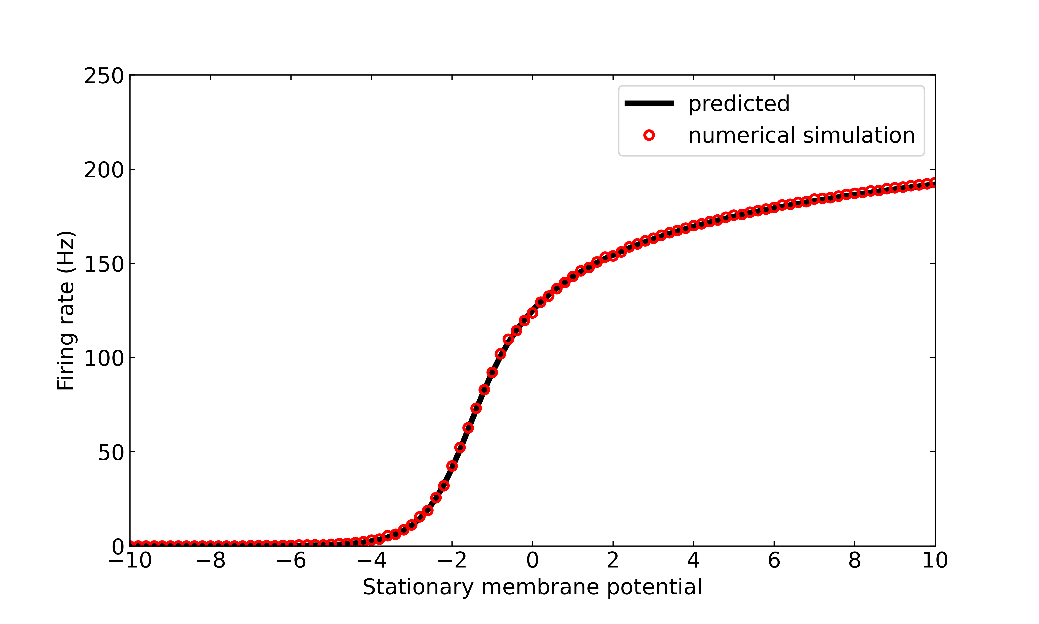


**c**


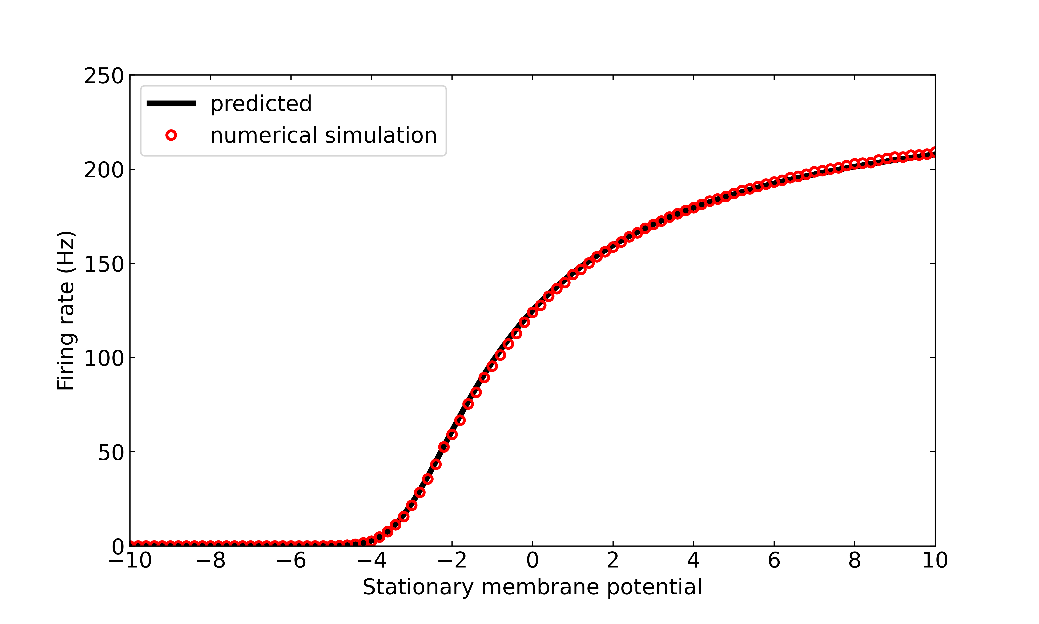


**Figure S2.** Comparison of predicted $\rho\left( u_{s} \right)$, and numerical simulation of $\rho\left( u_{s} \right)$. (a) the Abstract neuron model (b) the Escaping LIF neuron model (c) the Diffusive LIF neuron model


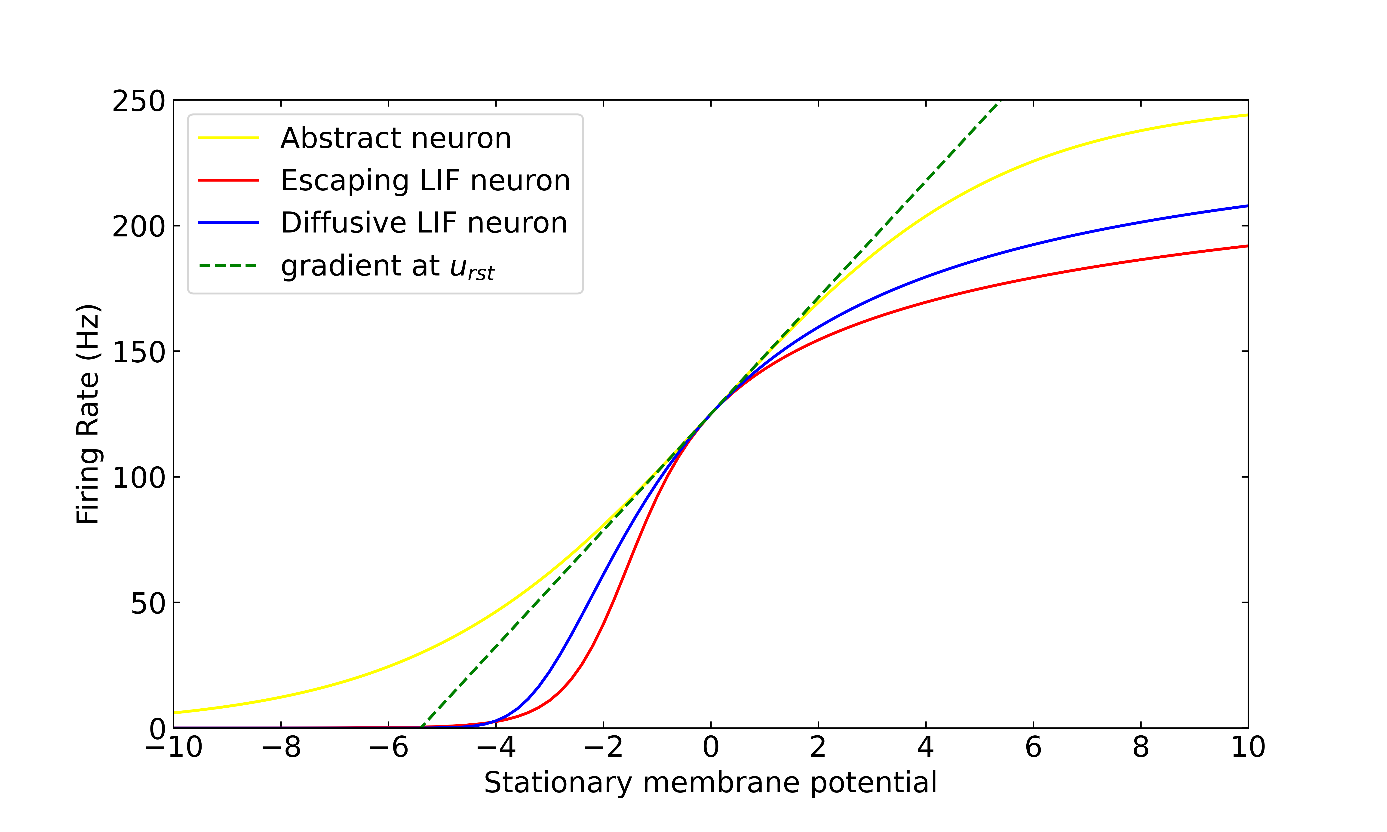


**Figure S3.** Comparison of $\rho_{A}\left( u_{s} \right)$ & $\rho_{E}\left( u_{s} \right)$ & $\rho_{D}\left( u_{s} \right)$


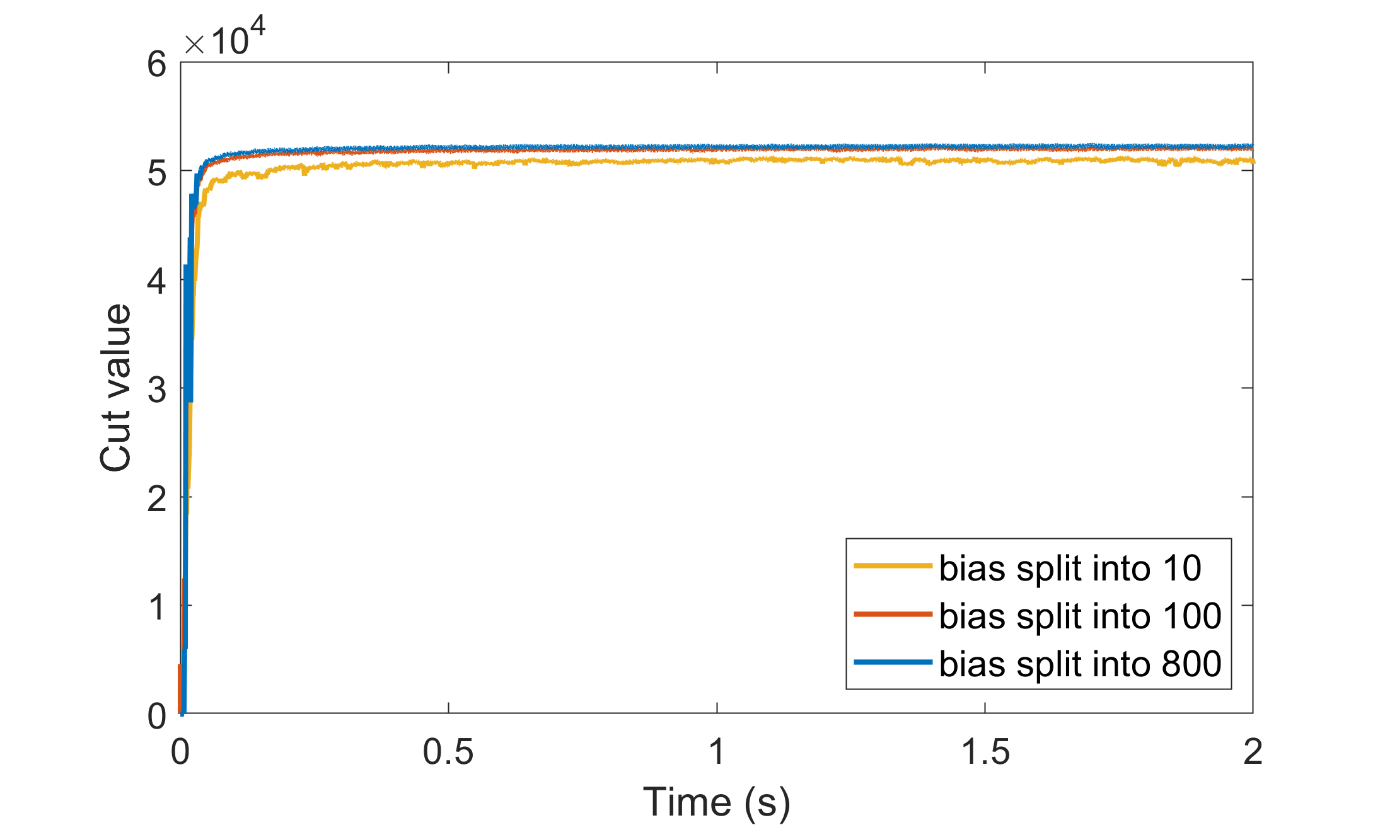


**a**


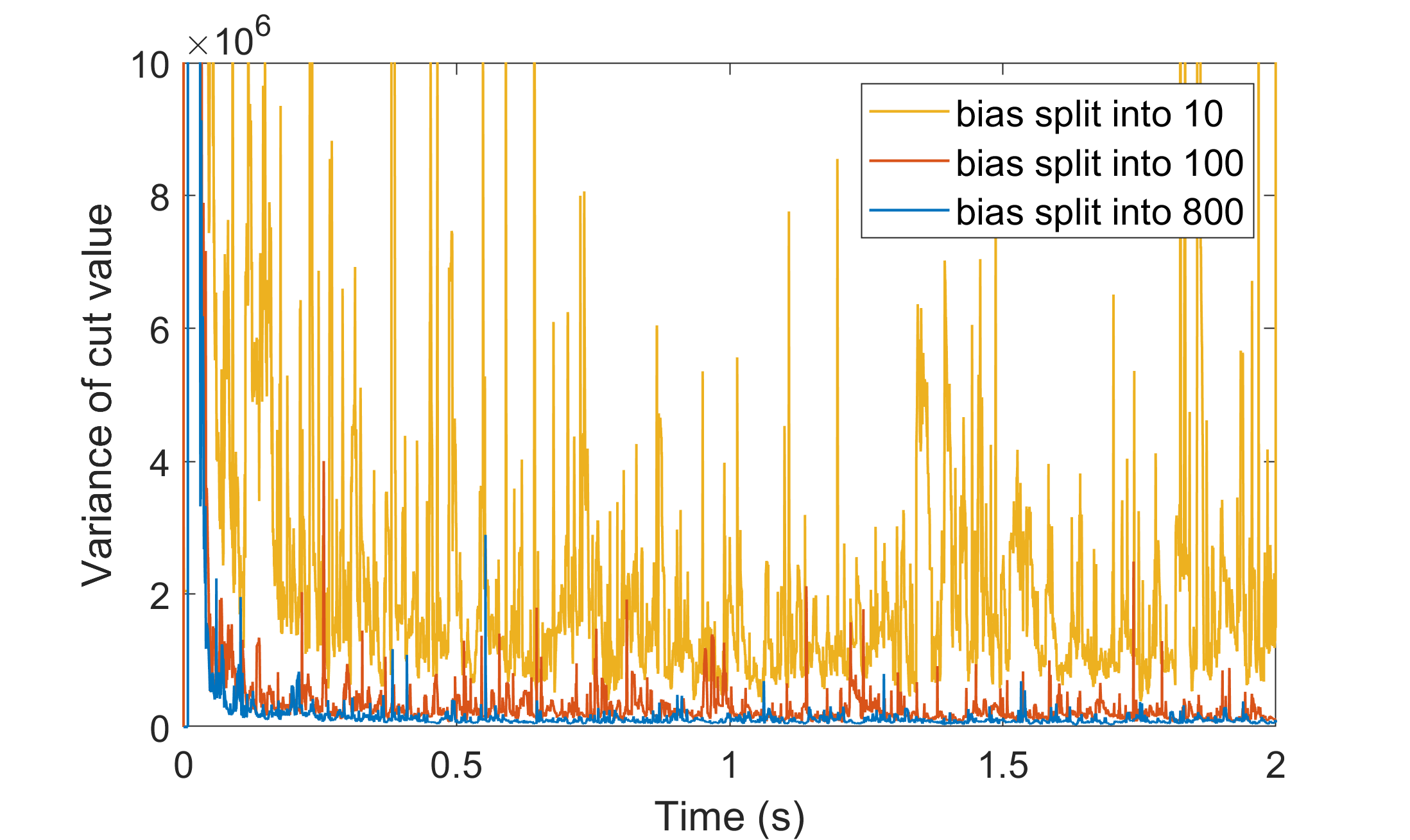


**b**

**Figure S4.** Simulation results of solving 200-node Max-Cut problem with 90% weight density with different numbers of bias neurons in bias split method. (a) the average cut values and (b) the average variances of cut values in the conditions of bias split into 10, 100 and 800 neurons. Each result is the average of 100 times.


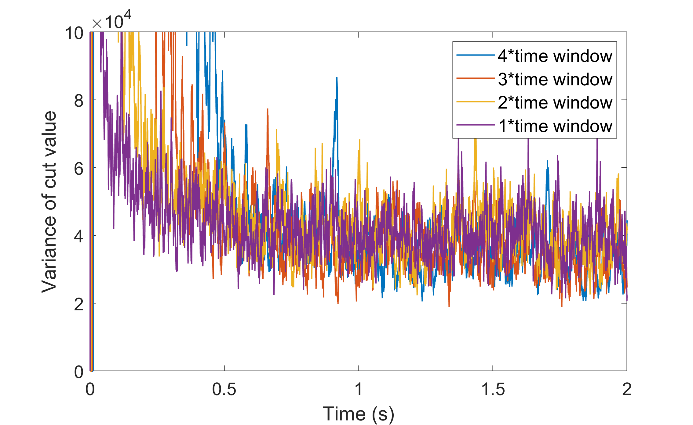

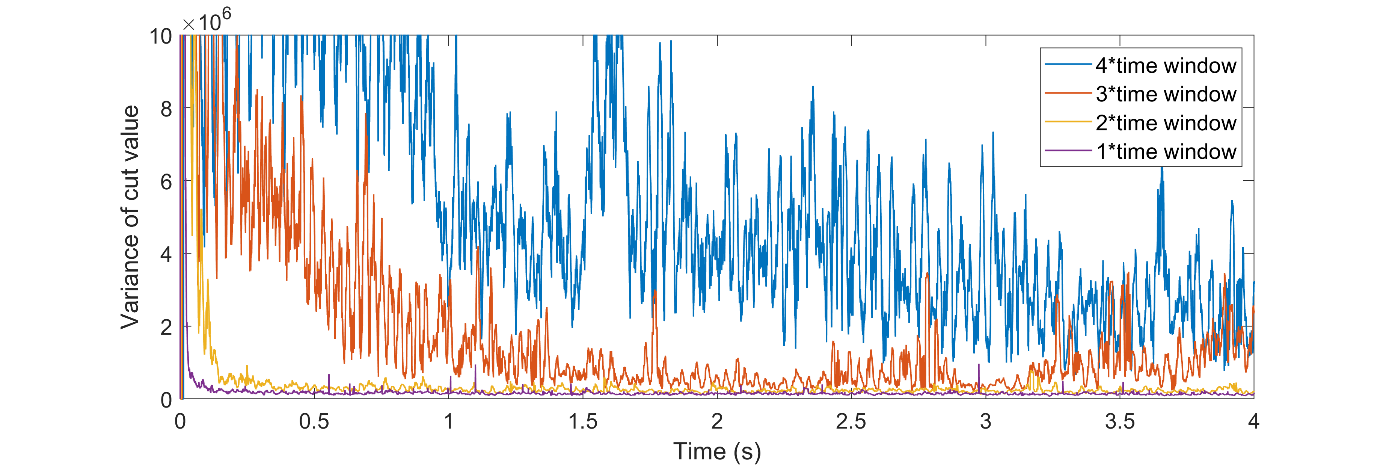

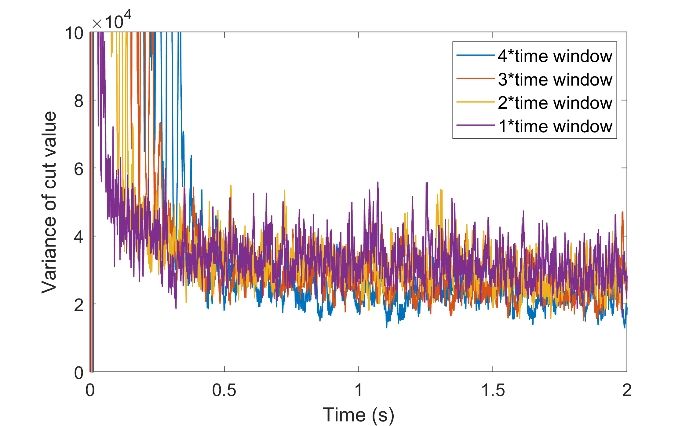

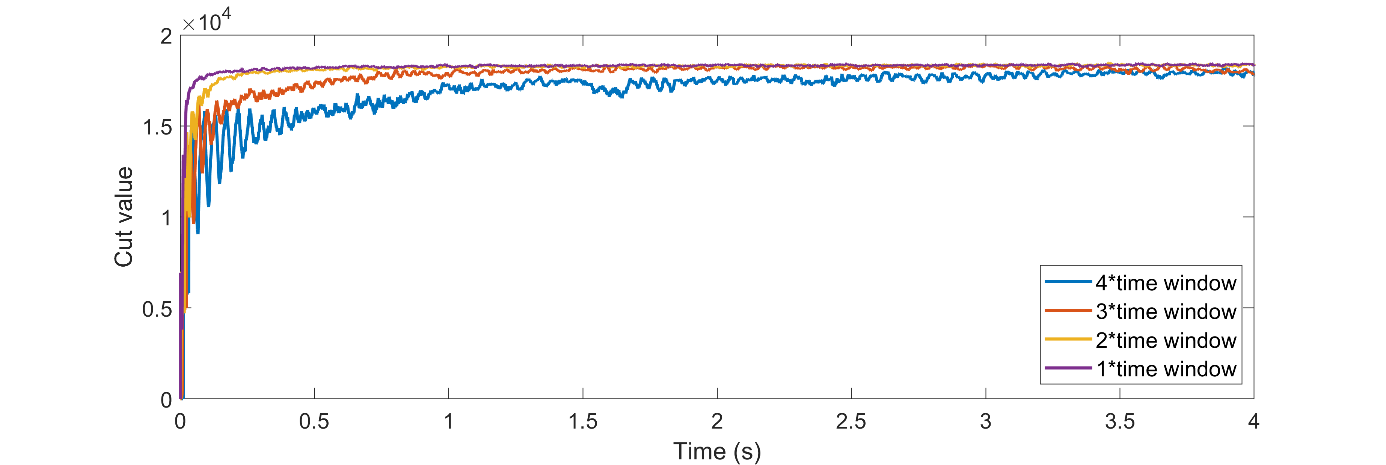

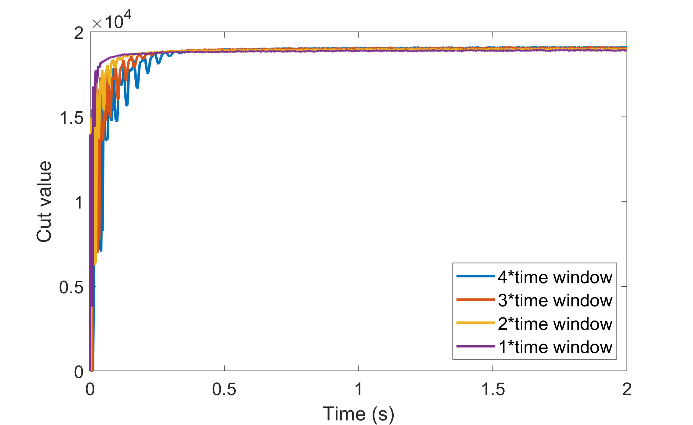

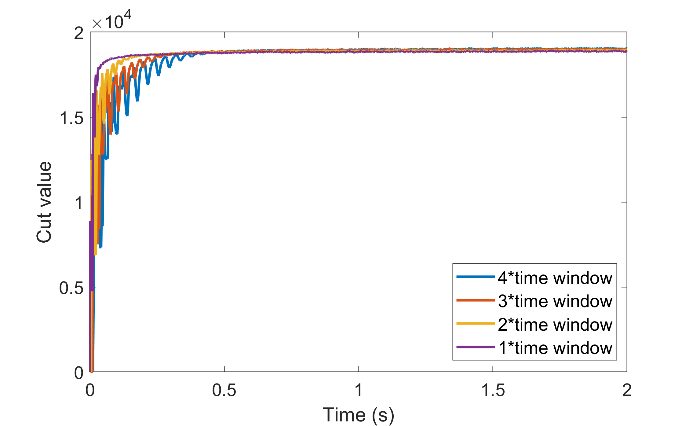
**Figure S5.** Simulation results of solving 200-node Max-Cut problem with 30% weight density using different time window lengths and criteria. (a-c) are the average cut values and (d-f) are the average variances of 100 times each. (a) and (d) are in the case of bias split into 800 bias neurons, (b) and (e) are in the case of bias split into 100 bias neurons, and (c) and (f) are in the case of bias split into 10 bias neurons.

**f**

**e**

**d**

**c**

**b**

**a**


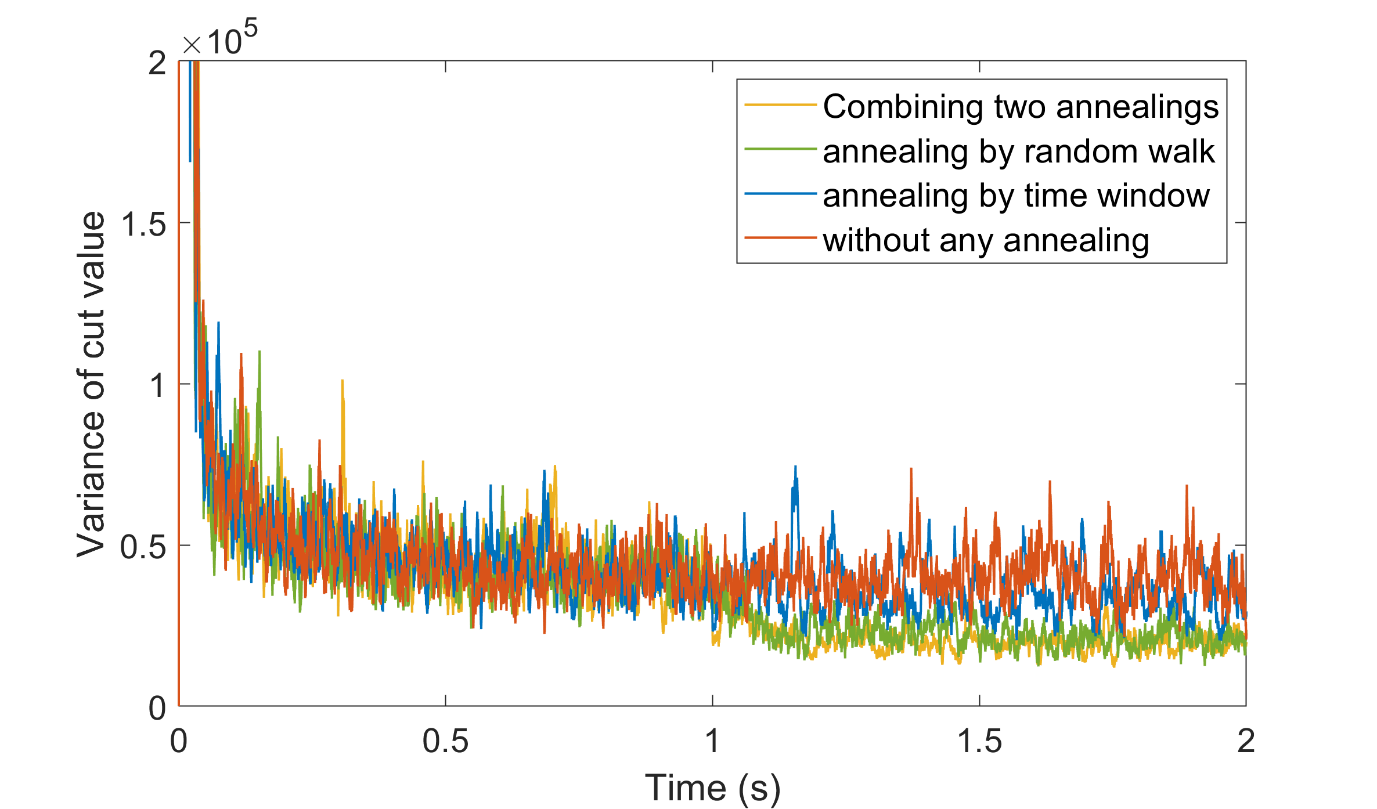


**Figure S6.** Average variances of cut value over 100 times corresponding to Figure 4f


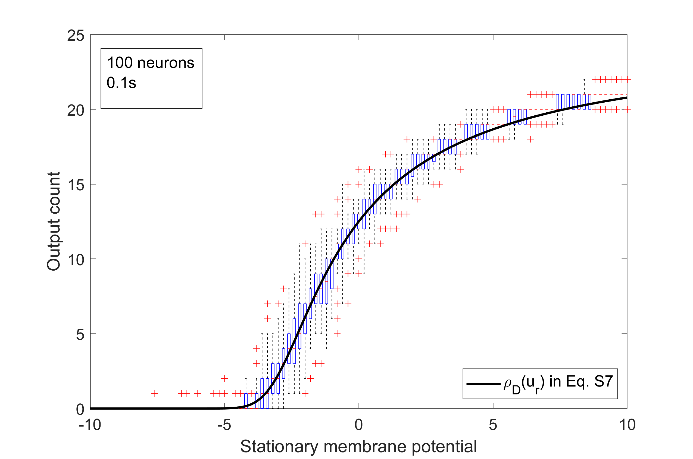

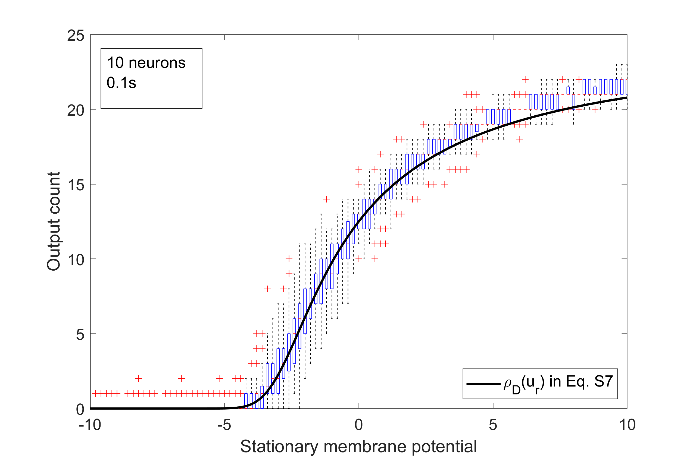


**b**

**a**


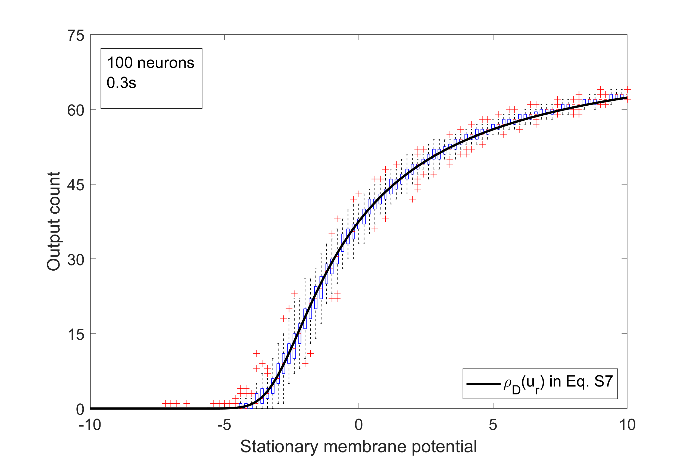

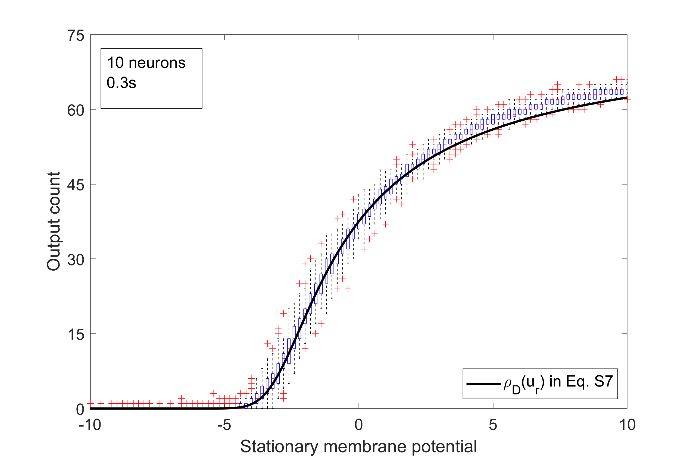


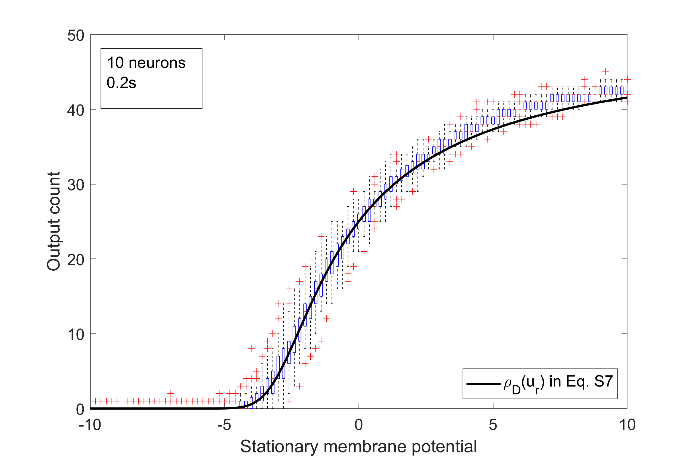

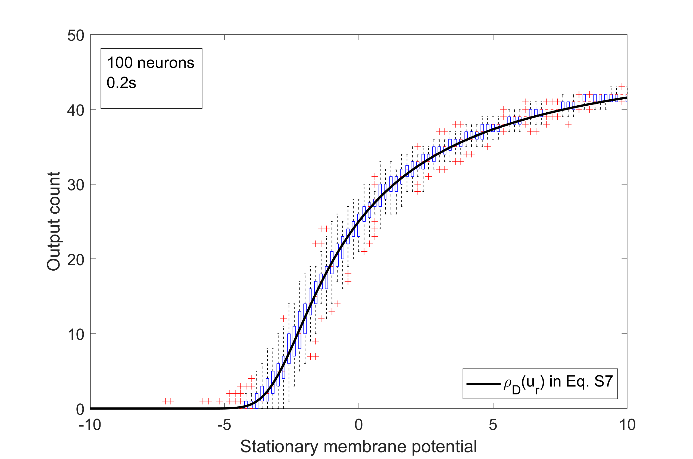


**f**

**e**

**d**

**c**


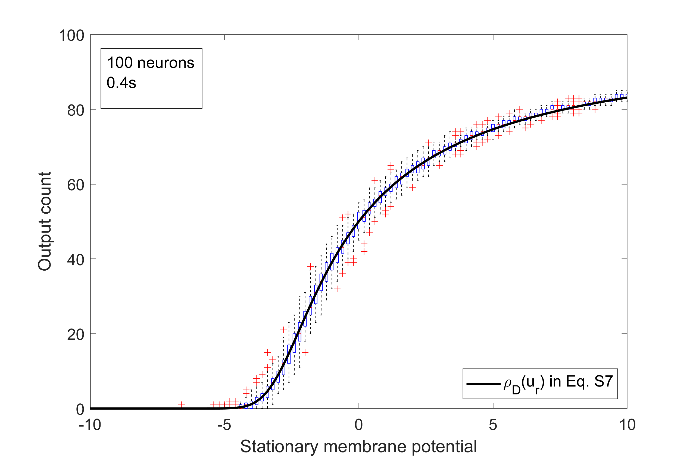

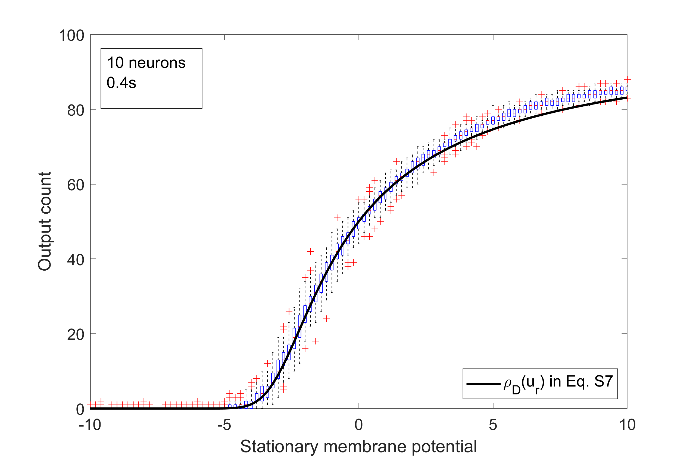


**h**

**g**

**Figure S7.** Variances of firing rate over different time with 100 results. (a), (c), (e) and (g) are results over 0.1s, 0.2s, 0.3s and 0.4s each with 10 neurons. (b), (d), (f) and (h) are results over 0.1s, 0.2s, 0.3s and 0.4s each with 100 neurons. Black lines in (a-h) are $\rho_{D}\left( u_{s} \right)$ from Equation (S7).


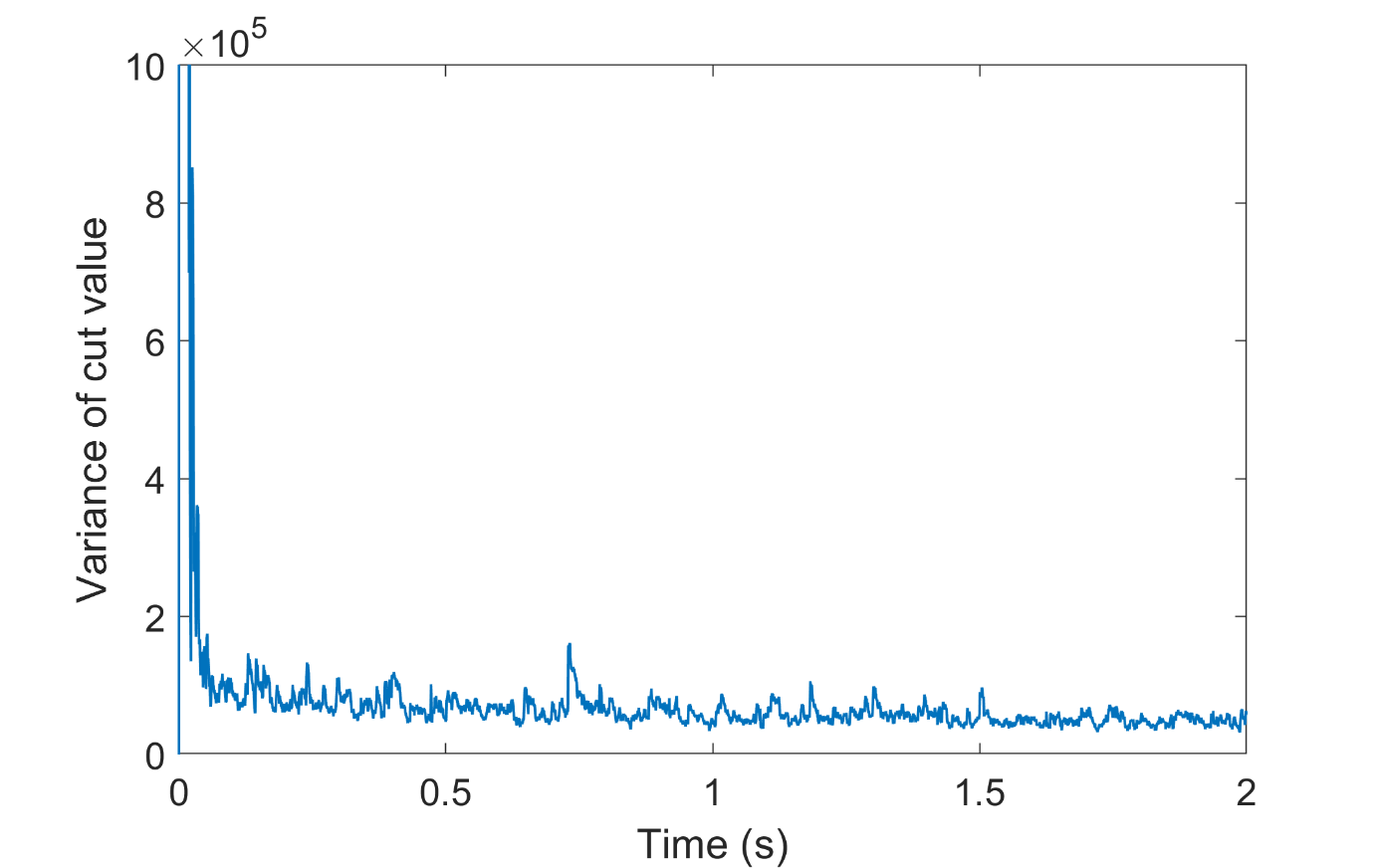
 **Figure S8.** Average variances of cut value over 100 times corresponding to Figure 4g


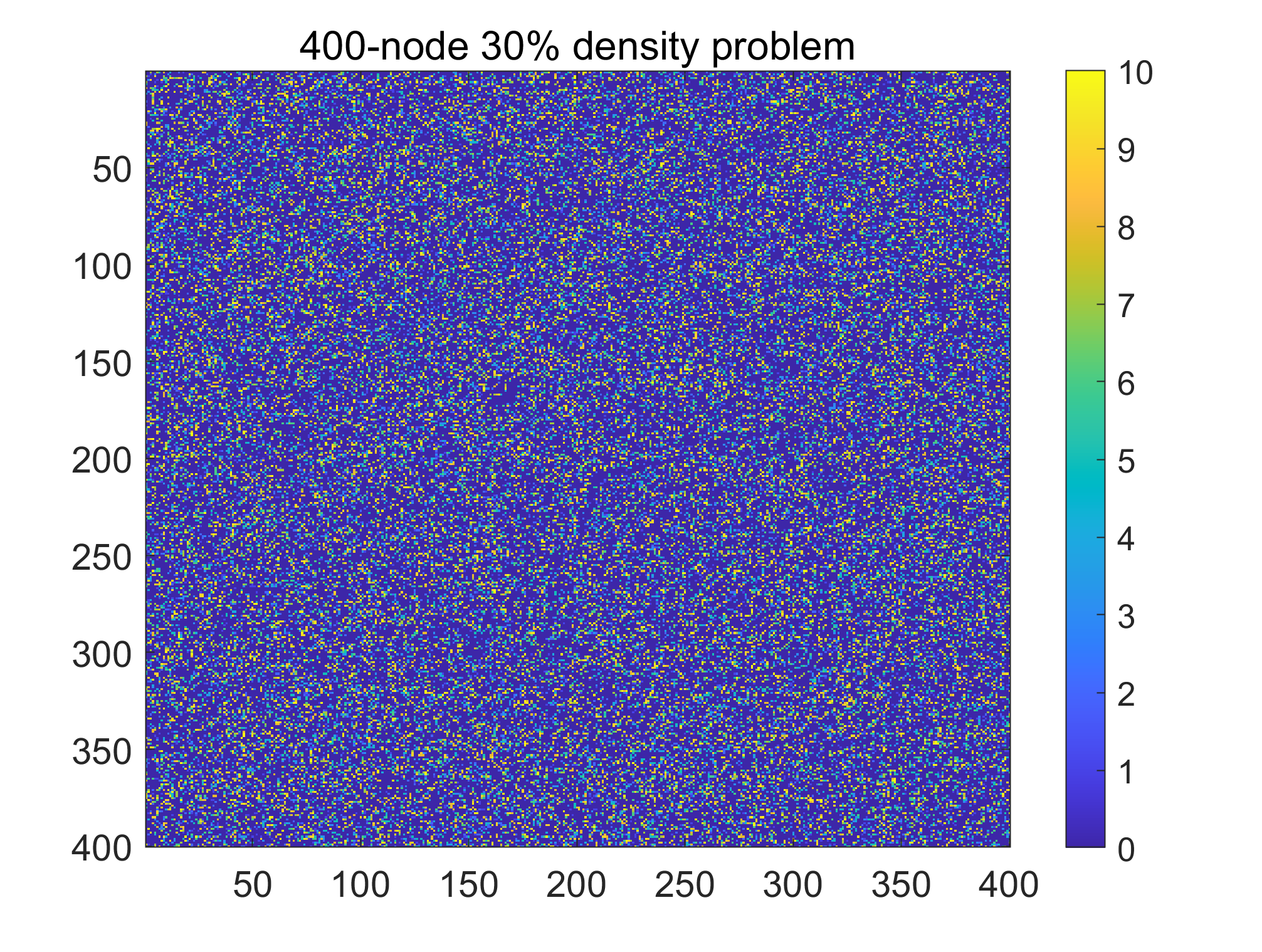

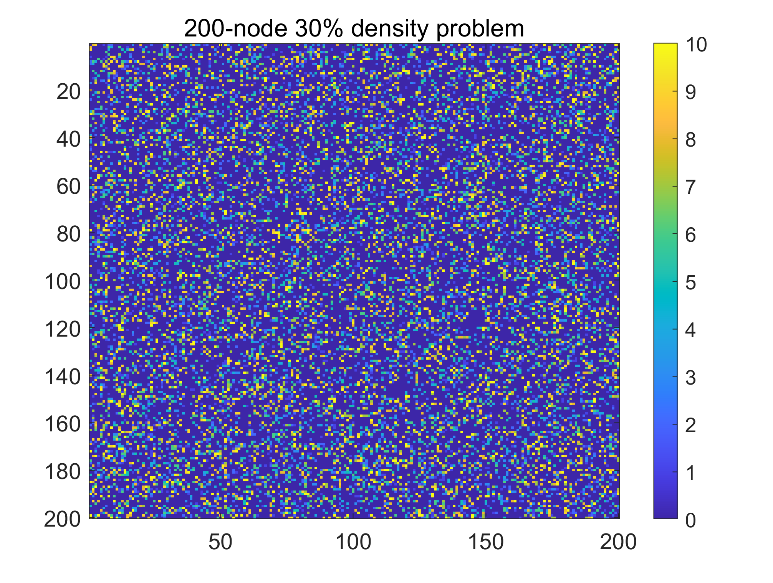

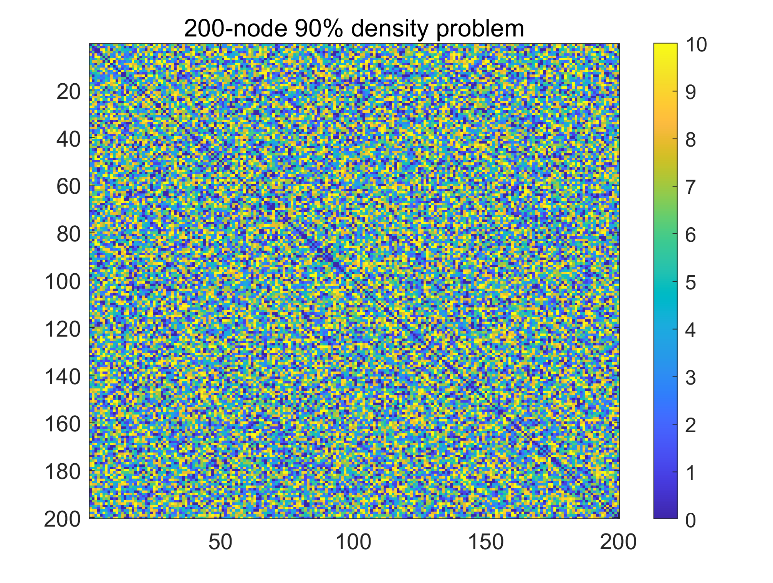

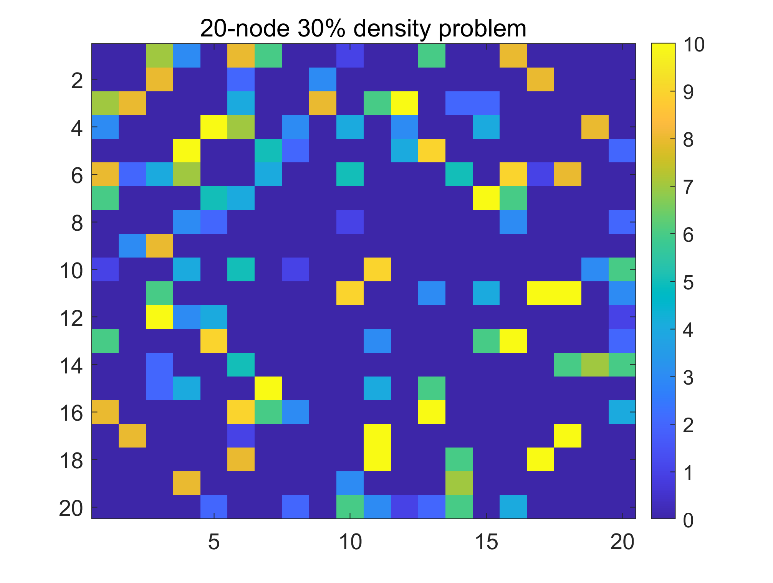

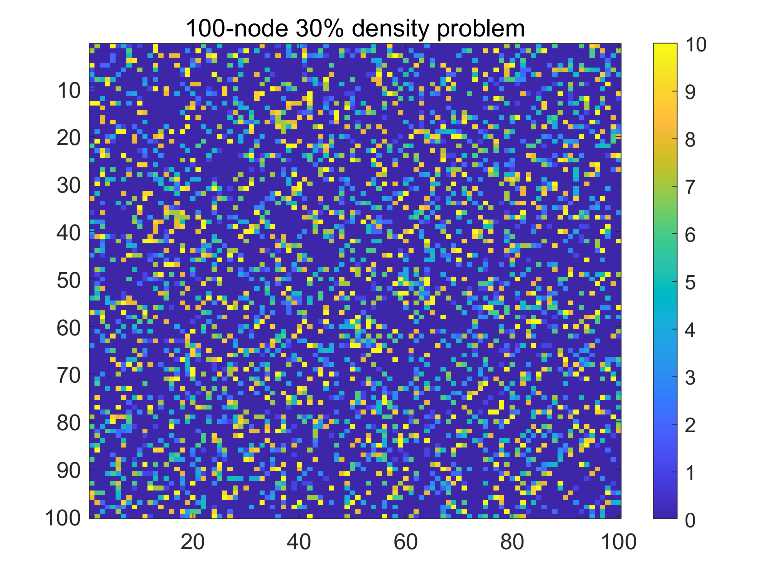


**e**

**d**

**c**

**b**

**a**

**Figure S9.** The Max-Cut problems used in simulations. (a) 100-node 30% weight density problem in Figure 2c. (b) 20-node 30% weight density problem in Figure 4c. (c) 200-node 90% weight density problem in Figure 4d-e. (d) 200-node 30% weight density problem in Figure 4f. (e) 400-node 30% weight density problem in Figure 4g
